# Resisting Resistance to Immune Checkpoint Therapy: A Systematic Review

**DOI:** 10.3390/ijms21176176

**Published:** 2020-08-27

**Authors:** Yolla Haibe, Ziad El Husseini, Rola El Sayed, Ali Shamseddine

**Affiliations:** Division of Hematology/Oncology, Department of Internal Medicine, American University of Beirut-Medical Center, Beirut 11-0236, Lebanon; yh25@aub.edu.lb (Y.H.); ze29@aub.edu.lb (Z.E.H.); re101@aub.edu.lb (R.E.S.)

**Keywords:** immune checkpoints, checkpoint inhibitors, resistance, overcome, mechanism of action, tumor micro-environment, canonical pathways, antigen presentation, regulatory cells

## Abstract

The treatment landscape in oncology has witnessed a major revolution with the introduction of checkpoint inhibitors: anti-PD1, anti-PDL1 and anti-CTLA-4. These agents enhance the immune response towards cancer cells instead of targeting the tumor itself, contrary to standard chemotherapy. Although long-lasting durable responses have been observed with immune checkpoints inhibitors, the response rate remains relatively low in many cases. Some patients respond in the beginning but then eventually develop acquired resistance to treatment and progress. Other patients having primary resistance never respond. Multiple studies have been conducted to further elucidate these variations in response in different tumor types and different individuals. This paper provides an overview of the mechanisms of resistance to immune checkpoint inhibitors and highlights the possible therapeutic approaches under investigation aiming to overcome such resistance in order to improve the clinical outcomes of cancer patients.

## 1. Introduction

We are finally reaching an era where we have hope for better treatment options for the management of metastatic cancer. It is a time where breaking the news to patients and their families is somehow less difficult with new means of therapy that are providing light at the end of the tunnel, with hopes of more long-term, maintained responses. However, no matter how optimistic we may be, results are not guaranteed. Not all patients respond, and resistance to such therapy exists. For this reason, understanding tumor biology and how such treatments work is important for finding means to optimize the therapeutic approach and improve the response.

The recently “apprehended” old understanding of tumor biology and cancer onco-pathogenesis has unveiled an entire spectrum of treatment possibilities, shifting focus from targeting tumor cells to targeting and mobilizing the immune system against malignancy. The concept has been there for quite a while, pioneered by Dr. William B. Coley in the 1890s [1], re-addressed via the cancer immune-surveillance theory proposed by Paul Ehrlich, and re-ascertained by Burnet et al. in the 1950s [2]; nevertheless, the concept did not reach implementation until the new millennium [3,4,5]. Tumorigenesis is no longer perceived as an odd event but rather a frequent happening that is usually suppressed by the host’s immunity [6,7].

Immune responses and dynamic interactions of immune cells with malignant cells as well as the surrounding tumor micro-environment lead to different consequences with different phases of immune-surveillance: elimination with the production of adaptive memory cells; equilibrium leading to immune tolerance; and finally; escape with consequent tumor growth [8]. Based on this concept, research has been focused on finding all the factors affecting these different phases. Furthermore, a better understanding and elucidation of the mechanisms of immune evasion and escape brought scientists one step closer to developing the means to overcome tumor growth and enhance immune stimulation to thereby improve patient responses to immunotherapy.

**Figure 1:**
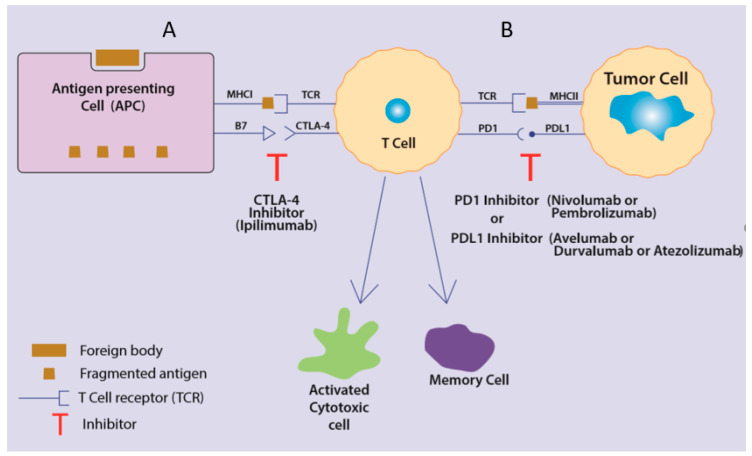
T-cell activation and inhibitors of checkpoint inhibition. (**A**) Antigen presenting cells introduce the fragmented antigens to foreign bodies presenting phagocytosed tumor cells to T-cells via major-histocompatibility complex I (MHC-I) and T-cell receptor (TCR) binding. This binding, along with co-stimulation by B7/CD28 receptor interaction, causes T-cell activation and further differentiation into activated cytotoxic cells and memory cells. Moreover, this process is hindered by an inhibitory checkpoint or CTLA-4 that takes the place of CD28 in binding to B7, thereby blocking activation. Ipilimumab is an inhibitor of CTLA-4 that can restore activation. (**B**) When activated, the cytotoxic T-cell requires the interaction of its TCR with the MHC-II present on the tumor cell for response. Nevertheless, this too is blocked by yet another inhibitory checkpoint or programmed cell death protein-1 (PD-1) present on T-cells with its correlating ligand PDL-1 on the tumor cells. Inhibitors of either PD-1 or PDL-1 can be used to reverse this blockage and allow the killing to proceed.

The mechanism by which the host’s immunity approaches malignant cells is a multi-step immune cell–tumor cell interaction, where host antigen presenting cells (APCs) activate naïve host T-cells against presented tumor antigens via the binding of major histocompatibility complex-I (MHC-I) present on APCs with T-cell receptors (TCR) present on T-cells [9]. Full activation usually requires co-stimulatory signals by the binding of receptors such as CD28 and B7 found on T-cells and APCs, respectively [10]. On the other hand, such a step is usually regulated by inhibitory checkpoints in order to avoid excessive stimulation, auto-immunity and self-tissue damage [11,12]. Of these inhibitory check-points, the most studied and implemented in practice are the cytotoxic T-lymphocyte-associated protein 4 (CTLA-4), programmed cell death protein-1 (PD-1), and programmed cell death protein ligand-1 (PDL-1) (Figure 1). Ipilimumab, an example of a CTLA-4 inhibitor, has been approved by the FDA since 2011 to be used in malignant melanoma [13], with approvals for multiple other malignancies having followed, such as renal cell carcinoma (RCC), non-small cell lung cancer (NSCLC), head and neck squamous cell carcinoma, urothelial carcinoma, lymphoma, hepatocellular carcinoma, and all microsatellite instability-high tumors and others. Remarkable responses have been noted, mostly when ipilimumab has been combined with other checkpoint inhibitors, anti-PD1/PDL-1 inhibitors specifically [14].

Multiple antibodies blocking the PD-1/PDL-1 inhibitory axis have been established, such as pembrolizumab, nivolumab and cemiplimab as PD-1 inhibitors, and durvalumab, atezolizumab and avelumab as PDL-1 inhibitors. They are known to act as inhibitors of the inhibition of immunity, and they unleash activated tumor-reactive T-cells in a various number of malignancies [15] (Figure 1).

Despite remarkable durable responses to immunotherapy with corresponding survival benefit in multiple approved indications, these responses to check-point blockade are very heterogeneous and not consistent among different patients. Some patients respond initially then lose benefit, having “acquired resistance”, while others do not respond at all, having “primary innate resistance” [16].

Two of the major challenges in optimizing cancer immunotherapy and improving patient outcomes are understanding the complex mechanisms entailing resistance, both innate and acquired, and determining countermeasures to improve response. Whether cancer-cell related, immune-cell related or micro-environment related, researchers have looked into these different mechanisms of resistance encompassing antigen presentation, cytotoxic T-cell activation and trafficking, as well as the stimulation of the immune-inhibitory axis. Means to either overcome such mechanisms of resistance or bypass them through various measures of immune activation have been studied on the cellular, enzymatic and molecular levels.

The goal of this review is to elaborate those mechanisms of resistance to immunotherapy and elucidate the strategies that can contribute to overcoming and bypassing this resistance. In this way, perhaps we can transform immunotherapy-resistant tumors into immunotherapy-sensitive tumors, where more and more patients can benefit from such treatment in the long-term in the clinic.

## 2. Mechanisms of Resistance to Immunotherapy and Ways to Overcome Them

**Figure 2:**
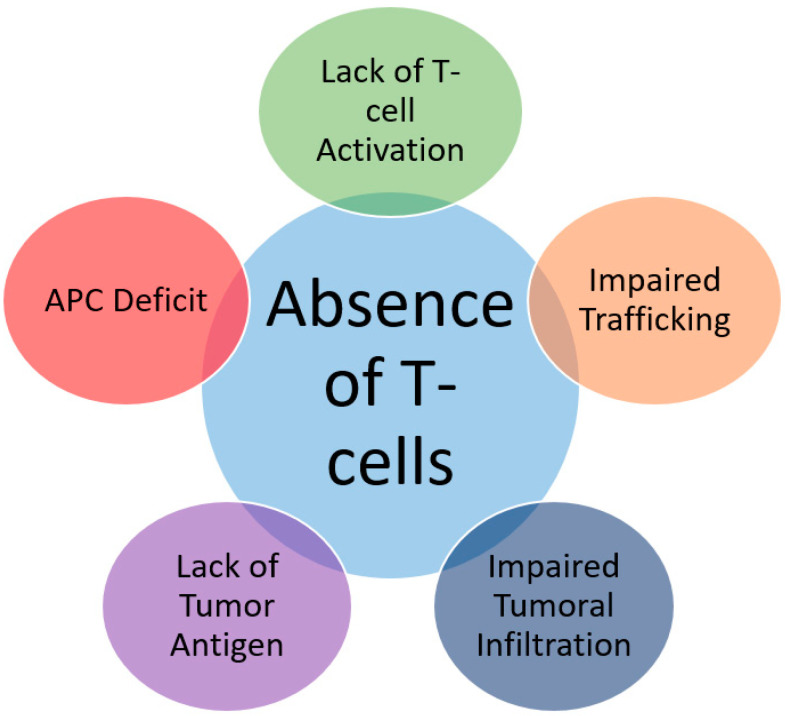
Etiologies of T-cell deficiency in tumor micro-environment.

Multiple intrinsic and extrinsic factors take part in tumor cell evasion from immune activation at the genetic, enzymatic and cellular levels. These factors affect the presence of effector T-cells in the tumor micro-environment either via defective or absent antigen presentation, an APC deficiency, and poor T-cell trafficking or infiltration (Figure 2). Mechanisms of resistance are not only tumor-cell related—in other words, “intrinsic”—but are also immune-cell and micro-environment related, and they are referred to as “extrinsic” mechanisms of resistance [17].

In brief, intrinsic resistance is when cancer cells alter processes related to:

(1) Immune recognition via the alteration of antigen presentation, such as the downregulation of MHC-I, for example [18];
(2) Cell signaling by affecting the mitogen-activated protein kinase (MAPK) [19] and interferon gamma pathways, for instance [20];
(3) Gene expression such as the expression of PTEN [21,22], JAK2 [23], EZH2 [24] and PI3K/AKT mutations [25], leading to T-cell exhaustion;
(4) The DNA damage response [26];
(5) The expression of various different checkpoints in secondary resistance settings to halt T-cell activation, including LAG-3, TIM-3, BTLA, TIGIT, VISTA, etc. [27].

On the other hand, extrinsic resistance occurs external to tumor cells through changes in T-cell activation. This involves regulatory T-cells (Tregs) [28], myeloid derived suppressor cells (MDSCs) [29], tumor-associated macrophages (TAMs) [30] and multiple canonical pathways [31] affecting the percentage of tumor infiltrating lymphocytes (TILs) and influencing the cytokine profile and cell toxicity [32].

With deepened insights into these mechanisms of resistance, many strategies are being developed to overcome such resistance and enhance anti-tumorigenic T-cell function, mostly in a combination of multi-modal approaches. These approaches target increasing tumor immunogenicity, overcoming T-cell exhaustion, improving the tumor micro-environment and enhancing tumor infiltration by activated T-cells. (Table 1).

### 2.1. Inadequate T-Cell Infiltration due to Lack of Tumor Antigens and Absence of Antigen Presentation

#### 2.1.1. Absence of Antigenic Protein

The recognition of cancer cells by CD8+T-cells through the presentation of tumor antigens as foreign is the first step in the induction process for the adaptive immune response (Figure 1). High neo-antigen and tumor burden loads are the backbone of the anti-tumor response [5,33]. There are three types of tumor antigens. First, tumor specific antigen (TSA) is only found in tumor cells [34]. It is comprised of viral antigens: antigens derived from oncogenic viruses, usually expressed on virally induced tumors and targeted by the immune system in the case of virus-associated tumors [35]. For example, in cervical carcinoma, peptides derived from oncoprotein E7 of human papillomavirus 16 (HPV16) are used to stimulate CD8+ lymphocytes [36,37].

TSA also includes different mutation-associated neo-antigens that play an important role in the CD8+ T-cell recognition of tumor cells after treatment with immune checkpoint blockade. Mutation-associated neo-antigens are usually overexpressed in tumors with high tumor mutational burden (TMB), which is defined as a high expression of mutations in cancer cell DNA and is considered as a promising biomarker for predicting responses to checkpoint blockade [38,39]. Second, there is the cancer germline antigen, found specifically in male trophoblastic and germ line cells. Finally, there are tumor associated antigens, usually overexpressed in tumor cells, such as Her-2 protein, weakly expressed in normal tissues [35,40].

Multiple solid tumors respond differently to immune checkpoint inhibitors due to heterogeneity in TMB and neo-antigen expression, which vary significantly from one tumor type to another. Studies conducted by Van Allen et al. as well as Rizvy et al. demonstrate that a high TMB and elevated neo-antigen expression were significantly correlated with responses to anti-PDL1 treatment in NSCLC and to anti-CTLA-4 treatment in melanoma [41,42,43]. By contrast, pancreatic cancer and prostate cancer, well known for their low TMB, were more resistant to anti-PD1/anti-PDL1 or anti-CTLA-4 therapy when compared to other solid tumors with high TMB [5].

Any mechanism contributing to the loss of expression of tumor antigens can lead to acquired resistance to checkpoints inhibitors. Anagnostou et al. [44] showed a decrease in neo-antigen expression in relapsed NSCLC patients after treatment with immune checkpoint inhibitors. Acquired resistance to immune checkpoint inhibitor treatment in tumors with high TMB was attributed to the process of immune-editing, where there has been a selection for low-immunogenicity sub-clones and increased intra-tumoral heterogeneity, harboring mostly clones that lack neo-antigen expression [45].

Moreover, although the high expression of tumor antigens is important for T-cell response induction, it is not the major limiting factor, as per a study conducted by Spranger et al. The argument focused on the important role other factors play in T-cell recruitment regardless of antigen expression. For example, they found that despite a similar adequate number of neo-antigens and proper tumor antigen presentation in both cold (non-T-cell-inflamed tumor) and inflamed tumors, non-T-cell-inflamed melanoma lacked any T-cells [46]. In addition, RCC with a known relatively low mutational burden has shown great responses to checkpoint inhibitors, emphasizing the presence of other factors at play in the response to immunotherapy [47].

One way to overcome the inadequate T-cell infiltration due to a lack of tumor immunogenicity is supplying the patient with T-cells that target the antigens with low immunogenic potential, and this is possible by generating, ex vivo, a large amount of CD8+ T-cells designated to target a specific tumor antigen, such as NY-ESO-1 in sarcoma and MART-1 in melanoma, through adoptive cell therapy (ACT). These generated T –cells are re-infused to the patient to target a specific antigen [48,49,50]. Another approach is the creation of mutational neo-antigens via vaccinations, stimulating dendritic cells (DCs) to produce activated T-cells against tumor antigens [51,52]. The combination of ACT or tumor vaccines with anti-PDL-1 treatment may be a plausible mechanism for overcoming the lack of expression of neo-antigens in certain tumors, thus generating an improved targeted response toward tumor cells [53,54].

Another possible approach to overcoming the lack of antigen expression may include the use of de-methylating agents such as histone deacetylase inhibitors and DNA methyl-transferase inhibitors (DNMTi), as epigenetic drugs that can potentially play an important therapeutic role in tumors with low antigen burden, by increasing tumor antigen expression and inducing endogenous retroviruses [55,56].

#### 2.1.2. Absence of Antigen Presentation

##### Beta_2_-Microglobulin (β_2_m)

The MHC-I molecule is composed of an α chain and a non-covalently associated β_2_m protein. Any loss of the β_2_m protein leads to the failure of tumor antigen presentation on the MHC-I molecules, thereby promoting the escape of tumor cells from cytotoxic CD8+ T-cells. Mutations in β_2_m were seen in melanoma cases; some tumors had primary resistance, while others acquired the resistance after initially responding to the immune checkpoint inhibitors [57,58]. A potential way of overcoming the resistance caused by the loss of β_2_m is by using natural killer (NK) cells. NK inhibitory receptors recognize MHC class I-positive cells and produce a signal to inhibit lysis. Hence, NK cells have an inborn capacity to target MHC class I-negative cells. Adoptive NK cells should be considered in the treatment of autologous β_2_m-deficient cancer cells [59].

##### Transporters Associated with Antigen Processing (TAP)

Peptides generated from tumor-associated antigens are translocated to the endoplasmic reticulum by the TAP, where they are packed with HLA class I molecules and transported to the cell surface. Deficiencies in TAP lead to defects in HLA class 1 antigen presentation. In vitro studies have shown that deficiencies in TAP and HLA class I in malignant melanoma cells can lead to escape from the immune response [60]. Moreover, a downregulation of TAP and HLA class I was associated with tumor progression in breast carcinoma [61]. In a mouse model, an infection with a flavi-virus upregulated the expression of MHC-I despite a deficiency in TAP, but the experiment did not work with human cells [62]. To date, no clear technique has been discovered for overcoming the deficiency in TAP; hence, more studies and theories are needed.

##### Pattern Recognition Receptors

To mount an immune response, APCs such as DCs and B-cells express surface receptors known as pattern recognition receptors (PRRs). PRRs are composed of five families responsible for detecting and interacting with pathogen-associated molecular patterns (PAMPs) and damage-associated molecular patterns (DAMPs). Mounting an immune response requires the internalization of these targets for display by MHC-I for T-cell recognition [63,64,65,66]. The activation of PRRs consequently induces cytokine production and the upregulation of stimulatory molecules to promote the cross-priming and co-stimulation of innate immune cells, mainly DCs and macrophages [67,68]. The lack of presence of DAMPs can lead to a deficiency in CD4+ T-cells due to a decrease in the maturation of DCs and an increased production of immunosuppressive factors [69,70]. Vachelli et al. highlighted the importance of the presence of the protein Formyl Peptide Receptor-1 (FPR-1) on DCs in breast and colon cancer, as its absence leads to a decrease in overall survival due to a lack of anticancer immune response because of the failure of antigen presentation and hence the failure of T-cell activation [71].

PRR agonists, activators of phagocytosis and antigen presentation by APC in the tumor micro-environment (TME) are currently being studied for overcoming resistance to immune checkpoint inhibitors. Shekarian et al. was able to demonstrate that the use of PRRs agonists is a possible tool for overcoming resistance to anti-PDL1 and anti-CTLA-4 in a preclinical model [72].

Immune checkpoint inhibitors have shown limited activity in certain tumor types like breast, prostate and pancreas [73,74]. Recently, a combination of radiation or chemotherapy therapy with immunotherapy has been “a la mode”.

Radiation therapy (RT), used in the treatment of different types of cancer, induces DNA damage leading to cell death [75,76]. RT, through increased neo-antigen formation and PDL-1 upregulation, has been found to have a synergistic effect when combined with checkpoint inhibitors for the treatment of solid tumors, leading to tumor regression as well as improved systemic and local tumor control [77,78,79].

Novel combination modalities have been under investigation to try to overcome resistance to checkpoint inhibitors by using RT. Demaria et al. conducted a study on mice with poorly immunogenic metastatic cancer types where the models were treated either with a single modality regimen using only anti-CTLA-4 blockade or a combination of anti-CTLA-4 and radiation therapy. The combination group showed a statistically significant improvement in survival compared to that receiving the single modality therapy [80]. Furthermore, Dewan et al. suggested that the use of a fractionated dose of radiation therapy may cause an immune-mediated abscopal effect when combined with anti-CTLA-4 inhibitors; however, to date, the abscopal effect is still controversial, and no studies have been able to prove the efficacy of this concept [81]. Many other clinical studies are ongoing to try to overcome immune resistance by combining RT and immune checkpoint inhibitors.

For several years, chemotherapy has been considered as an immunosuppressive treatment. It was not until very recently that certain chemotherapeutic agents were found to increase anti-tumor immunity by causing immunogenic cell death and by interfering with the mechanisms used by tumors to evade the immune system [82]. During cell death induced by chemotherapy, tumor antigens will be released into the tumor micro-environment, leading to the emission of DAMPs. Some chemotherapeutic agents such as anthracyclines play a role in activating the expression of PRR toll-like receptor [83]. Moreover, chemotherapy can increase the sensitivity of tumor cells to T-cells through granzyme-B- and perforin-dependent pathways. It can increase antigen presentation by enhancing the expression of MHC class I and leads to the strengthening of T-cell activity by downregulating co-inhibitory molecules or upregulating co-stimulatory molecules [84].

### 2.2. Genetic T-Cell Exclusion and Insensibility to T-Cells

T-cell infiltration within the TME is a crucial step in the process of the immune recognition of foreign bodies for stimulating an immune response. T-cell exclusion mediated by the MAPK pathway, WNT/B-catenin pathway, and loss of expression of PTEN plays an important role in the mechanism of resistance to immune checkpoint inhibitors.

#### 2.2.1. MAPK Oncogenic Signaling

The MAPK pathway plays an important role in cell proliferation and apoptosis. An increase in this signaling pathway inhibits the recruitment and function of T-cells by producing inhibitory substrates such as VEGF (vascular endothelial growth factor), interleukin (IL)-8 and other inhibitory cytokines [85]. Increased MAPK signaling will contribute to T-cell exclusion and immune evasion as per a study conducted by Loi at al. that showed immune evasion in patients with triple negative breast cancer with MAPK pathway activation [85,86]. The use of MAPK inhibitors in mouse models has been shown to induce an increase in PDL-1 levels, tumor cell infiltration and MHC-I expression, with a consequent increase in tumor cell cytotoxicity [87,88].

The combination of BRAF and MEK inhibitors, used to target the MAPK pathway, with anti–PD-1/PD-L1 has been shown to increase anti-tumor immunity and decrease exhaustion and apoptosis. Studies using BRAF and MEK inhibitors in combination with anti-CTLA-4 were stopped at early phases due to an increase in toxicity [87,88,89,90].

#### 2.2.2. Tumor Suppressor Phosphatase and Tensin Homolog (PTEN)

PTEN is a tumor suppressor that downregulates the activity of the phosphatidyl-inositol 3-kinase (PI3K) pathway, promoting the proliferation and survival of tumor cells. The loss of PTEN is associated with lower CD8+ cytotoxic T-cell infiltration, as well as the upregulation of VEGF and PI3K, leading to increased tumorigenesis [21,91]. Moreover, the loss of PTEN and activation of PI3K lead to increased PD-L1 gene expression, which contributes to immune resistance [92]. It was reported that non-T-cell-inflamed tumors have a higher frequency of PTEN mutations compared to T-cell-inflamed tumors [16]. Selective PI3K inhibitors were found to hinder the growth of melanoma cells in vitro. Subsequently, in vivo studies were performed showing that the combination of a PI3K inhibitor with an anti-PD-1 inhibitor is superior to monotherapy. Combining the two therapies enhanced tumor growth inhibition and led to improved survival [21].

#### 2.2.3. WNT/Beta-Catenin Signaling Pathway

The WNT/beta-catenin pathway is an oncogenic pathway involved in promoting oncogenesis and increasing the invasive metastatic potential of tumor cells [93]. Activation of the WNT/beta-catenin pathway leads to a decrease in CD103+ DC infiltration by suppressing the expression of CCL4, which is responsible for DC attraction, eventually decreasing T-cell activation [94]. A study conducted by Spranger et al. showed that the degree of infiltration of CD8+ T-cells in tumors is inversely correlated with beta-catenin activation in tumor cells in mouse models with melanoma. Tumor cells with pathway activation showed poor responses to immune checkpoint inhibitors contrary to tumor cells without beta-catenin mutations [94]. The SCOOP Study, which is an ongoing phase I/II trial in metastatic colon cancer, is investigating the safety and efficacy of combining pembrolizumab with BBI608, a downregulator of WNT/β-catenin signaling; the preliminary results are promising [95].

#### 2.2.4. Vascular Endothelial Growth Factor (VEGF)

VEGF is secreted by tumor cells and is found in abnormally high concentrations in patients with cancer. This elevated level induces angiogenesis and stimulates tumor growth. Moreover, it was found that VEGF induces immunosuppression through the downregulation of DCs, decreased activity and recruitment of CD8+ T-cells and stimulation of the expression of different checkpoints such as PD-1, TIM-3 and CTLA-4 on activated cytotoxic T-cells, leading to resistance to immune checkpoint inhibitors [96,97]. Furthermore, VEGF is associated with the expression of Fas ligand on tumor endothelial cells that kill effector but not regulatory T-cells [98].

Anti-VEGF has been combined with multiple therapies in attempts to overcome resistance. The combination of anti-VEGF with ACT impeded vascularization and improved survival in mouse models [99]. When combined with anti-CTLA-4 inhibitors in phase I clinical trials in metastatic melanoma patients, the synergy with anti-VEGF was shown to improve the disease control rate as well as the median overall survival as compared to single agent ipilimumab [100]. Other studies are ongoing in metastatic renal cancer and melanoma patients [101].

#### 2.2.5. Transforming Growth Factor-β (TGF-β)

TGF-β is a regulatory cytokine that prevents tumor progression by regulating the differentiation and proliferation of tumor cells. Tumor cells can evade the inhibitory effects of TGF-β by inactivating its receptor or preventing the downstream cascade [102]. Moreover, TGF-β is found to downregulate cytotoxic T-cells while promoting their differentiation into regulatory T-cells. In the TME, cancer-associated fibroblasts are found to be the major producers of TGF-β, which is correlated with immune checkpoint inhibitor resistance [103]. 264RAD is a human monoclonal antibody targeted against αvβ6 integrin that was found to inhibit the downstream activation of TGF-β. Treatment with 264RAD in vivo was associated with a reduction in pharyngeal carcinoma and metastatic lung lesions [104]. Additionally, GC1008 (Fresolimumab) is an anti-TGF-β human monoclonal antibody that was tested in a phase I trial for safety in patients with melanoma and renal cell carcinoma [105]. In a mouse model of metastatic urothelial cancer, combining anti-PD-L1- and TGF-β-blocking antibodies facilitated T-cell infiltration into the TME with resultant tumor regression [106]. Finally, LY3022859 is monoclonal antibody targeted against TGF-β receptor that was assessed in a phase I trial, where no safe dose was reached for a phase II trial [107].

#### 2.2.6. Indoleamine 2,3-Dioxygenase (IDO)

IDO is a cytosolic enzyme that catalyzes tryptophan degradation, and tryptophan deficiency leads to T-cell cycle arrest. IDO is found to suppress the immune reaction either by activating regulatory T-cells or by suppressing effector T-cells. While some tumor cells produce IDO constitutively, IFN-γ can stimulate the production of IDO in tumor cells or APCs [108,109]. 1-methyl-tryptophan (1MT) can inhibit IDO and induce a T lymphocyte-dependent immune response in murine models [108]. In mouse models with IDO expression, anti-CTLA-4 inhibitors showed no effect on tumor growth. When combining anti-CTLA4 with 1MT, there wasa a significant increase in survival [108]. Currently, a phase 2 trial is studying the combination of an IDO inhibitor, indoximod, with checkpoint inhibitors in patients with metastatic melanoma (NCT02073123, clinicaltrials.gov). Moreover, a phase I trial is investigating the combination of a peptide vaccine that targets IDO with anti-CTLA-4 inhibitors in patients with non-resectable melanoma [110].

#### 2.2.7. Interferon-γ Receptor Pathway

Interferon-gamma (IFN-γ) is released by activated T-cells after interaction with tumor antigens. It activates the Janus kinases, JAK1 and JAK2, leading to anti-tumor outcomes. Loss-of-function mutations in JAK1/2 lead to unresponsiveness to the IFN-γ signal, with a decrease in signal transducer and activator of transcription 1 (STAT1) phosphorylation. This leads to a decrease in PD-L1 and MHC-I expression. This pathway was found to be involved in both primary and acquired resistance [23,111]. In an effort to overcome it, a tumor cell-based vaccine was created to induce a cytotoxic immune response. This vaccine was associated with IFN-γ -induced PD-L1 upregulation in the TME. Finally, the combination of anti-PD-1 and this cell-based vaccine induced a regression in the melanoma mouse model where the anti-PD-1 alone failed [112].

#### 2.2.8. Enhancer of Zester Homolog-2 (Ezh2)

Ezh2, involved in the intrinsic and extrinsic mechanisms of resistance, is a histone methyl transferase that is highly expressed in many tumors and plays an important role in tumor cell proliferation, invasion, epithelial mesenchymal transition and metastasis; hence, it is associated with poor prognosis [113,114]. Ezh2 aids in downregulating Th1-type chemokines that subsequently decrease cytotoxic T-cell tumor infiltration [115]. Zingg et al. reported that an increase in Ezh2 expression was noted in melanoma cells after treatment with anti-CTLA-4, due to an increase in the production of tumor necrosis factor alpha (TNF-α). This leads to a decrease in antigen presentation, a decrease in the immunogenicity of the TME and, therefore, resistance to treatment by immune checkpoint inhibitors [116]. Inhibiting Ezh2 expression enhances the activity of anti-CTLA-4 treatment and suppresses tumor growth. In vivo, the combination of GSK503, an Ezh2 inhibitor, with anti-CTLA-4 or IL-2 proved to be more efficient than monotherapies in the treatment of melanoma [116].

### 2.3. Impairment of T-Cell Functionality by Immunosuppressive Signaling Receptors

Just as the infamous CTLA-4, PD-1 and PDL-1 were found to have a significant effect on T-cell activation against malignant cells, other checkpoints have also been studied. These include both stimulatory and inhibitory checkpoints, summarized in Table 2 and further elaborated on in the text. In this part, we discuss the impact of inhibitory or immunosuppressive signaling receptors that hinder anti-tumor immunity by impairing T-cell function.

#### 2.3.1. Cytotoxic T-Lymphocyte-Associated Protein 4 (CTLA-4)

CTLA-4 receptors are usually found on T-cells. They act at the level of lymph nodes in early immune activation [117]. They were found to compete with CD28 co-stimulatory receptors in binding to B7 on APCs, thereby impeding immune activation [118]. Ipilimumab was the first antibody blocking CTLA-4 approved for the treatment of patients with cancer [119,120]. When compared to standard treatment with chemotherapy, an increase in overall survival was noted with the use of anti-CTLA-4, especially in patients diagnosed with RCC, melanoma and NSCLC [121,122].

#### 2.3.2. Programmed Cell Death Protein-1 (PD-1) and Programmed Cell Death Protein Ligand-1 (PDL-1)

PD-1 and PDL-1 are a later-stage inhibitory checkpoint and its receptor, respectively. Later-stage means that their role is mostly in the subsequent stages of the T-cell response, at the level of peripheral tissue [117]. They represent an essential part of adaptive immune resistance [123,124]. The expression of PD-1 and PDL-1 can be constitutive or induced. Constitutive expression is usually dependent on some oncogenicity-inducing factors or pathways such as MYC, KRAS, STAT3, PTEN, etc. Inducible expression usually relies on secreted inflammatory factors such as IFN-γ, TNF-α, IL-6, IL-8 and others [125]. Inhibitors of PD-1 and PDL-1 have been widely used in a number of malignancies with very good responses. However, a lot of patients are either inherently resistant or develop resistance along the way, as is elaborated within this review. Combining PD-1/PDL-1 blockade with agents affecting factors involving their expression has proven to be of utmost value in enhancing their therapeutic potential [88,126,127,128,129].

Furthermore, after receiving monotherapy with anti-PD1 or anti-PDL-1, some patients were found to develop rapid tumor progression, named as hyper-progressive disease (HPD). The tumor growth rate (TGR), which estimates the tumor volume expansion over time, is compared between the baseline imaging and the post- immune checkpoint inhibitor (ICI) treatment imaging, where HPD is described as the doubling of the tumor size (TGR ≥ 2) [130,131]. Multiple biomarkers have been associated with HPD. Human murine double minute 2 (MDM2) is a negative regulator of the of the tumor suppressor p53; hence, with its amplification, p53 is degraded [132]. From the six patients with MDM2/4 amplification, four had hyperprogressive disease with more than 2-fold increases in tumor size post treatment with anti-PD-1/PD-L1 [133]. It is hypothesized that PD-1/PD-L1 inhibitors induce the amplification of MDM2 through the JAK-STAT pathway (previously described). Therefore, a possible approach to overcoming HPD is combining MDM2 inhibitors with ICI [131,132]. Additionally, it could also be secondary to the proliferation of regulatory T-cells in the tumor micro-environment secondary to PD-1/PD-L1 blockade [134].

Patients diagnosed with HPD after treatment with nivolumab (anti-PD-1) had significantly higher levels of absolute neutrophil count (ANC) and C-reactive protein (CRP) within 4 weeks of treatment initiation [135]. The frequency of HPD differed between different cancer types, reaching 21%, 9% and 29% in non-small cell lung cancer, melanoma and head and neck squamous cell carcinoma, respectively [134]. Moreover, in the randomized phase III trial, CheckMate057, nivolumab resulted in a higher risk of death during the first 3 months secondary to disease progression than docetaxel [136]. No difference was noted between anti-PD-1 and anti-PD-L1 monotherapies [130], while no cases were reported with anti-CTLA-4 monotherapy [134]. In a retrospective review of patients treated with anti-PD-1/PD-L1 in phase I trials, the TGR was calculated and correlated to overall survival (OS). HPD was associated with older age and worse OS while not being associated with higher tumor burden at baseline [130]. Finally, Ferrara et al. compared in a retrospective study the frequency of HPD in patients with non-small cell lung cancer treated with anti-PD-1/PD-L1 or chemotherapy and showed that it is more common with PD-1/PD-L1 inhibitors but can still occur with chemotherapy treatment [137].

#### 2.3.3. Lymphocyte Activation Gene-3 (LAG-3)

LAG-3 is a trans-membrane protein expressed on regulatory T-cells and activated CD4+ and CD8+ T-cells. LAG-3 functions as a negative cellular regulator that binds MHC-II and is upregulated on exhausted T-cells. In murine models, the combination of anti-LAG-3 and anti-PD-1 led to an improvement in the CD8+ T-cell response [12,138]. Moreover, a soluble LAG-3 immunoglobulin (Ig) has been studied in a phase I trial in advanced RCC and in combination with chemotherapeutic agents, gemcitabine and paclitaxel, in advanced pancreatic adenocarcinoma and metastatic breast carcinoma, respectively. In addition, in a phase I trial, soluble LAG-3 Ig in combination with a peptide vaccine has been studied in advanced melanoma [12]. Finally, there is a randomized phase I/II trial assessing the safety and effectiveness of anti-LAG-3 with and without anti-PD-1 therapy (NCT01968109), still ongoing.

#### 2.3.4. T-Cell Immunoglobulin Mucin-3 (TIM-3)

TIM-3 is an immune regulator that can induce cell apoptosis or exhaust the immune system. It is found to be upregulated on exhausted cytotoxic T-cells along with PD-1. Moreover, cytotoxic T-cells with both TIM-3 and PD-1 expression were found to be the most abundant non-cytokine-producing lymphocytes, with an inability to secrete IL-2, TNF-α and IFN-γ [139]. Shohei et al. showed that TIM-3 upregulation takes place during the inhibition of the PD-1/PD-L1 pathway with checkpoint inhibitors, leaving the tumor resistant to immunotherapy [140]. In vivo, the mice that progressed after anti-PD-1 therapy were treated with anti-TIM-3, and survival was improved significantly from 5 weeks to 11.9 weeks [140].

#### 2.3.5. V-Domain Ig Suppressor of T-Cell Activation (VISTA)

VISTA is a negative immune regulator that is mainly expressed in hematopoietic tissues. It is found in the TME and stimulates the conversion of naïve T-cells into Tregs. Moreover, VISTA suppresses the activity of cytotoxic T-cells, and reduces the production of IL-10, TNF-α and IFN-γ. It is responsible for acquired resistance against immune checkpoint inhibitors [141,142]. In murine models, anti-VISTA antibodies suppressed the growth of the present tumor and decreased Tregs, but anti-VISTA monotherapy was not curative. In an effort to overcome this acquired resistance, anti-VISTA antibodies were combined with a peptide-based cancer vaccine. This therapeutic combination was shown to significantly inhibit tumor growth and increase survival in mouse models [142].

#### 2.3.6. T-Cell Immuno-Receptor with Ig and ITIM Domains (TIGIT)

TIGIT is a co-inhibitory receptor of the Ig superfamily correlated with CD28. It is mainly found on NK cells, exhausted T-cells (effector, helper and memory) and Tregs [143,144,145]. TIGIT usually binds two types of ligands, CD155 and CD112, which are expressed on APCs and T-cells, competing with CD226, thereby inhibiting the activation of T and NK cells [146]. CD226, similarly to B7-CD28 binding, causes the co-stimulation of T and NK cells. Therefore, TIGIT, with high affinity to CD155, acts either by directly inhibiting co-stimulation through competitively binding to these CD226 substrates or through binding to CD226 receptors themselves and disrupting their homo-dimerization and function. In addition, TIGIT–CD155 interaction was found to lead to the increased tolerance of DCs by the inhibition of IL-12p40 production and induction of IL-10. It blocks the NK cell’s function via signal transductions through the PI3K-MAPK pathways as well by the limitation of NF-κB (nuclear factor-κB) signaling. Moreover, TIGIT may affect the TCR signaling pathway and consequently block productive T-cell activation. Many other mechanisms of action of TIGIT have been described in the literature, including the stimulation of anti-apoptotic molecules such as Bcl-xL; the upregulation of receptors for IL-2, IL-7 and IL-15 responsible for T-cell survival; and promoting and maintaining T-regs via Foxp3, as well as shifting the cytokine balance away from Th1 and Th17 by inhibiting the production of IFN-γ and IL-17 while enhancing Th2 cell cytokines [147,148,149].

In brief, TIGIT blockade may have a role in restoring T-cell and NK cell activation while decreasing tumor tolerance. Therefore, in cases of immunotherapy failure, theoretically, if TIGIT blockers are used concomitantly, they may improve outcomes. Such results have been portrayed in a few preclinical models and recently in clinical models with good results. In a study of patients with melanoma, TIGIT blockers used with PD-1 inhibitors improved CD8+ TIL proliferation, cytokine production, and degranulation [150]. Similarly, in another murine model, the same combination showed synergy, with restored cytotoxic T-cell function and tumor regression [12,151]. Most recently, preliminary results of the phase 2 CITYSCAPE trial of a TIGIT inhibitor tiragolumab combined with an anti-PDL1 agent atezolizumab demonstrated better efficacy, as discussed in the 2020 American Society of Clinical Oncology Virtual Scientific Meeting. There was notable improvement in the overall response rate, 37% versus 21% in favor of the combination versus the single agent atezolizumab, as well as improvement in progression-free survival for the combination when compared to the single agent atezolizumab (5.6 versus 3.9 months; hazard ratio, 0.58) [152].

#### 2.3.7. B and T lymphocyte Attenuator 4 (BTLA-4)

B and T lymphocyte attenuator (BTLA) is yet another receptor in the Ig supergene family [153] that is expressed not only by naïve and activated B- and T-cells but also by monocytes and DCs [154]. It binds to a receptor on tumor cells and tumor-related endothelial cells called herpesvirus entry mediator (HVEM), which is in fact part of the TNF family [155]. Upon the interaction of BTLA and HVEM, bi-directional signaling takes place, where BTLA-expressing cells are inhibited, with their proliferation and cytokine production halted, and HVEM-expressing cells, mainly TILs, are stimulated, with their survival potential enhanced [154,156,157,158]. This dual signaling is quite complex and depends on specific interactions of these receptors with variable mediators such as CD160 and LIGHT. Multiple studies have shown a significant role of BTLA blockade in boosting the T-cell response and anti-tumor immunity, when combined or not with PD1 inhibitors [27,159,160,161].

### 2.4. Lack of Stimulatory Checkpoints Promoting Immune Escape

#### 2.4.1. ICOS

Just as the CD28/B7-binding protein family has inhibitory checkpoints, it also includes stimulatory checkpoints, one of which is inducible co-stimulator, or ICOS, expressed mainly on T-cells, with its counterpart ICOS-ligand (ICOS-L) expressed by DCs, macrophages and sometimes tumor cells [162]. ICOS/ICOS-L interaction is responsible for the secretion of pro-inflammatory Th1-type cytokines that induce direct T-cell cytotoxicity [163]. ICOS basically assists TCR-mediated signal transduction by potentiating the PI3K–AKT–mTOR signaling cascade and increasing IL4 production [164]. The presence of ICOS+ lymphocytes and ICOS-L+ cells was found to have different prognostic value in various tumor settings [165,166]. Nevertheless, in general, ICOS stimulation enhances T-cell activation, and its use has been proven to synergize with and potentiate the activity of CTLA-4 [167,168] as well as PD-1 inhibitors [169]. Further research is ongoing to explore the role of ICOS stimulation as a means of re-activating the immune response following treatment with checkpoint inhibitors [170].

#### 2.4.2. OX40

OX40—another stimulatory transmembrane receptor found on CD4+ and CD8+ T-cells as well as NK cells, neutrophils [171,172] and Tregs [173]—is part of both innate and adaptive immunity. When it was bound to its ligand on APCs, IFN-γ levels were noted to be increased, thereby enhancing T-cell activation and sustaining Th1-type responses [174]. Such interaction also increased PDL-1 expression, which implicates the probable synergism of OX40 stimulation and PDL-1 inhibition [175], especially when they are given sequentially [176]. Other cytokines are noted to be produced upon OX40/OX40-L interaction, sustaining lymphocyte survival, proliferation and memory [177,178]. From another point of view, OX40 stimulation has a direct effect on Tregs, causing their depletion and functional disruption, once again stimulating anti-tumoral responses [179].

Furthermore, monoclonal antibodies agonizing OX40 are being explored in a number of malignancies including sarcoma, melanoma and breast cancer [180,181], where they have been noted to have powerful synergy in combination with checkpoint inhibitors [182].

#### 2.4.3. Glucocorticoid-Induced Tumor Necrosis Factor Receptor (GITR)

Finally, Glucocorticoid-Induced Tumor Necrosis Factor Receptor (GITR) is, as the name implies, a receptor of the TNF family, found on Tregs, CD4+ and CD8+ T-cells, NK cells, eosinophils and macrophages. Its corresponding ligand is expressed by APCs as well as vessel endothelial cells [183,184]. The stimulation of GITR has been found to increase IL2 and IFN-γ production while at the same time upregulating the available receptors for those cytokines, therefore enhancing effector T-cell activation [185,186]. Concomitantly, GITR stimulation leads to FOXP3 suppression, leading to eventual Treg inhibition [187]. Humanized GITR agonists are being studied in combination with checkpoint inhibitors, with preclinical data showing encouraging results for therapeutic anti-tumor potential [170,188]. In one preclinical in vivo study with melanoma in mouse models, GITR agonists have been shown to alter intra-tumoral Treg accumulation, with a greater ratio of effector T-cells to Tregs, suggesting greater effector T-cell activity and function [189]. In one phase I trial, MK-4166, a humanized IgG1 agonist monocloncal antibody, was tested as a single agent and in addition to pembrolizumab in patients with advanced solid tumors. MK-4166 was shown to be relatively safe with tolerable adverse events, with an objective response rate reaching 69% when combined with checkpoint inhibitors in immunotherapy-naïve melanoma patients [190].

### 2.5. Immunosuppressive Cells within the Tumor Micro-Environment

#### 2.5.1. Tumor Associated Macrophages (TAMs)

Tumors have the capacity to switch macrophages in their micro-environment into an immune-suppressive instrument that the tumor uses to invade, migrate and metastasize. Moreover, TAMs release the factors needed for angiogenesis and tumor growth. The growth factor colony stimulating factor 1 (CSF1) along with other cytokines, IL-4 and IL-13, promotes the immunosuppressive actions of TAMs [191]. Additionally, TAMs express suppressive ligands, such as B7-H4 and VISTA, that are the main mechanism of immunosuppression. These ligands work by downregulating cytokine production and inhibit T-cell activation and proliferation [192].

The blockade of CSF1 receptor (CSF1R) was noted to upregulate the immune checkpoints PD-L1 and CTLA4. In a mouse model of pancreatic ductal adenocarcinoma, anti-PD1 and anti-CTLA4 monotherapies induced inadequate responses. On the other hand, the combination of CSF1R blockade with either anti-PD1 or anti-CTLA4 initiated tumor regression [193].

#### 2.5.2. Regulatory T-Cells (T Regs)

The differentiation of naïve CD4+ T-cells into Tregs is induced by PD-L1 stimulation, where Tregs highly express CD25, CCR4, CTLA-4, PD-1 and PD-L1. PD-L1 and CTLA-4 function in peripheral tolerance by activating regulatory T-cells and inhibiting the effector T-cells [194,195]. In mice models, anti-CD134 and anti-CD25 monoclonal antibodies successfully reduced the number of regulatory T-cells within the peripheral lymphoid tissue and caused the regression of tumors [196,197]. Moreover, a complete response was only found in the combination of anti-CD25 and anti-PD-1 [196]. On the other hand, the combination of anti-PD-1 with anti-CD134 had a negative effect on the immune system and reduced survival in mouse models [198]. Studies conducted in rodents showed that targeting folate receptor 4 with monoclonal antibodies led to T reg depletion [197].

#### 2.5.3. Myeloid-Derived Suppressor Cells (MDSCs)

MDSCs present in the TME promote tumor growth, invasion and metastasis [199,200]. Meyer et al. studied the possible alteration in MDSCs in patients treated with ipilimumab by studying circulating MDSCs and found that a low presence of MDSCs in the TME correlates positively with response to checkpoint inhibitors. They hypothesized that MDSCs could be used as a predictive biomarker for the response to immune checkpoint inhibitors [201]. MDSCs were found to express elevated levels of PD-L1 that caused T-cell exhaustion in the tumor micro-environment [29]. In addition, these cells were found to be abundant in immunologically cold tumors, especially in glioblastoma that was found to be poor in cytotoxic T-cells and rich in T reg cells, MDSCs and TAMs [202].

Furthermore, Highfill et al. showed that the efficacy of checkpoint inhibitors was limited in rhabdomyosarcoma due to the accumulation of CXCR2+ MDSCs in the TME promoting immunosuppression and tumor growth. Hence, the disruption of CXCR2 will probably inhibit MDSC trafficking into the tumor bed and increase the response to anti-PD1 therapy [203].

#### 2.5.4. Innate Lymphoid Cells (ILCs)

Innate lymphoid cells (ILCs) are a family of lymphocytes that constitute a part of the body’s adaptive immunity. They co-exist and attain characteristics similar to those of T-cells and NK cells. ILCs have been found to be present in multiple mucosal and non-mucosal tissues in addition to in the peripheral blood. They are basically composed of five types of cells that are grouped into three sub-categories—ILC1, ILC2 and ILC3—depending on their receptor and cytokine profiles.

Extensive research has been conducted to study the role of ILCs in tumor growth, tumor suppression, immune surveillance and inflammation. They have been found to be very labile to cytokines released within the TME upon tissue damage. ILCs themselves have also been reported to secrete different chemokines and cytokines that play a significant role in tumorigenesis and immune surveillance. They can be both pro- and anti-oncogenic, and they can be plastic and revert from one form to the other depending on signals received from the surrounding micro-environment [204]. For example, IL-15-stimulated ILC1s promote tumor cell lysis via the production of IFN-γ and TNF-α, with the consequent upregulation of MHC-I expression, whereas IL-12-activated ILC1s have complete functional impairment and promote tumorigenesis. Moreover, ILC2s can induce cytotoxic T-cell recruitment in the presence of IL-33 through IL-5 production and eosinophilic stimulation. On the other hand, ILC2s were also reported to activate MDSCs via IL-13 and Tregs via IL-9, in addition to their promotion of tumor progression and migration by IL-4 and amphiregulin (AREG) production. Finally, ILC3s, when activated by IL-23, can secrete IL-17 as well as IL-22, which leads to T-cell immune-suppression [205], as has been shown in colon cancer models. Similarly to other sub-classes, ILC3s also have anti-tumoral activities described in lung cancer models, where it was hypothesized that tumor suppression takes place by the production of IFN-γ, resulting in the upregulation of adhesion molecules and the facilitation of the recruitment of other immune cells into the TME. Another form of ILCs studied in ovarian cancer models—the IL-22-producing NKp44– CD56+ TILs, which act as regulatory ILCs—inhibited the outgrowth of tumor-infiltrating CD8+ T-cells, with resulting worse clinical outcomes.

High expression of PD-1 was noted in progenitors of ILCs, most of which are lost during differentiation. Nevertheless, in a recent study in mice, PD-1 expression was noted to be upregulated in ILC2s in inflammatory situations [206], stimulated by IL-33. This event was noted to suppress cytokine release [207]. Moreover, it was hypothesized that PD-1 blockade with checkpoint inhibitors would therefore restore cytokine production, favoring cancer growth [208]. Other studies have also shown the presence of functional PD-1 receptors on ILC3s, with a suggested regulatory role, that can impact the tumor response to checkpoint inhibitors [209,210].

It seems clear that the TME, with its cytokines and variable interleukins, can have marked effects on the functions of ILCs, which themselves also have PD-1 receptors and can play a significant role in the tumor response. However, more research is needed to be able to control the mechanisms underlying these changes and how they affect tumor growth [211], in order to be able to implement these cells as a possible means of cultivating a proper immune reaction, facing the failure of checkpoint blockade treatment. Perhaps cytokine or interleukin agonists or antagonists may be used in synergy with checkpoint inhibitors [212].

### 2.6. Immune Metabolism and Resistance to Immunotherapy

Immune metabolism plays an important role in the immune response to malignancy. The dysregulation of immune metabolism can affect immune surveillance either at the cellular level or at the systemic host level, leading to lower ICI efficacy and primary or acquired resistance to immune checkpoint inhibition [213]. Dysregulation at the cellular level includes some of the mechanisms and pathways previously discussed, such as the VEGF, tryptophan and PI3K/AKT/mTOR pathways, or the stimulation of immunosuppressive cells such as TAMs or MDSCS and the inhibition of effector T-cells in the tumor micro-environment, as will be further elaborated on.

#### 2.6.1. Dysregulation of Immune Metabolism at the Cellular Level

The tumor micro-environment has proven to play a major role in the different phases of tumorigenesis. This TME, which is comprised of a large variety of different cell types in different metabolic states, has been proven to be integral in the shaping of the immune response to checkpoint inhibition. The cellular complexity of the TME seems to be very dependent on the limited metabolic nutrient availability over which immune cells, tumor cells and non-tumor cells compete [214]. Both tumor cells and activated cytotoxic T-cells as well as innate immune cells rely on aerobic glucose consumption. In cases of limited supply, cells use alternative metabolic means [215] to survive, such as the Warburg effect, with the shift of some immune cells from aerobic glycolysis to oxidative phosphorylation (OXPHOS) or fatty acid oxidation (FAO) [216,217]. These mechanisms have interestingly been shown to be related to immune checkpoint resistance via immune cell-related factors such as the impairment of effector T-cell activation or stimulation of T-regs and other tolerogenic DCs associated with immunosuppression.

##### Lactate

In cases of glucose shortage, high concentrations of lactate are produced, leading to the acidification of the TME. In acidic settings [218], VISTA is stimulated, leading to deficient cytotoxic T-cell function [219]. Furthermore, antigen presentation by DCs is impaired [220], and TAMs shift towards an M2 immune tolerant phenotype [221]; thereby, immune surveillance is blocked.

##### Hypoxia

The hypoxic conditions in the TME lead to the production and accumulation of hypoxia-inducible factor 1-alpha (HIF-1), which is a transcriptional regulator of the cellular response in hypoxic situations [222]. HIF-1 has been shown to promote immunosuppression via TAM suppression as well as the stimulation of PDL-1+ tumor-associated MDSCs and Tregs by FOXP3 upregulation [223,224,225].

##### Arachidonate Metabolism

Prostaglandins produced by cyclo-oxygenase 1 and 2 are inflammatory lipid mediators found in the TME [226,227]. They are released from membrane phospholipids through the arachidonate metabolic pathway. Prostaglandin E_2_ has been implicated in immune suppression [228]. A few pre-clinical trials have studied the combination of nonsteroidal anti-inflammatory drugs (NSAIDs) with checkpoint inhibitors in an attempt to act on the blockade of COX activity in the TME, and the results have been promising [229].

##### Pyruvate Kinase Muscle

In an attempt to provide substrates for aerobic glycolysis, tumor cells occasionally resort to the alternative splicing of pyruvate kinase muscle (PKM2) [230]. On the other hand, PKM2 has been proved in mice models to promote PDL-1 expression in cancer and immune cells, including innate cells as well as MSDCs [231,232,233].

##### Adenosine Pathway

Ectonucleotidase CD39+ and CD73+ are responsible for producing adenosine from adenosine-tri-phosphatase. Adenosine is found in high concentrations in cancer tissues and plays an important role in the progression of neoplasms by promoting the proliferation of immunosuppressive cells and inhibiting NK cells, CD8+ T-cells and DCs [234,235]. Allard et al. showed that the combination of a CD73 inhibitor with anti-PD1 can increase survival in mice by increasing CD8+ T-cells, since CD73-positive tumor cells are usually resistant to ICI [236]. Overman et al. recently showed promising results with the combination of Durvalumab with Oleclumab (anti-CD73) in pancreatic cancer patients [237]. Many trials using drug combinations with ICI are underway to try to overcome resistance to ICI.

#### 2.6.2. Dysregulation of Immune Metabolism at the Host Level

##### Obesity

Obesity is a complex disease characterized by excessive adipose tissue with dysfunctional adipokine signaling [238]. Obese patients usually have lower levels of the anti-inflammatory, anti-apoptotic peptide adiponectin [239] and higher levels of pro-inflammatory cytokines such as IL-6 and TNF [240], as well as higher levels of leptin. It was shown that obesity usually entails a chronic state of cytokine-driven low-grade inflammation [241], implicated in T-cell exhaustion, with a decrease in NK cells and CD8 T-cells but an increase in PDL1 expression [242]. Moreover, obesity was noted to promote MDSC recruitment, probably stimulated by high leptin levels [243,244].

It is well known from previous studies that a high BMI is usually associated with worse survival in cancer patients. However, a few recent studies have shown a positive correlation between BMI and survival, a phenomenon called the “obesity paradox” [245,246]. The “obesity paradox” was mostly noted with ICI treatment in metastatic and non-metastatic colorectal cancer and renal cell carcinoma [245,247], as it was hypothesized that fatty liver, usually found in obese patients, would delay the clearance of ICI by altering its metabolism in the liver [242,248], along with the aforementioned increased PDL-1 expression and chronic inflammatory status. One retrospective analysis of patients with NSCLC treated with ICI showed that longer overall survival was found in patients with an elevated BMI [249]. These studies had several limitations and were hampered by confounders.

##### Caloric Deficit

Caloric restriction improves immunosurveillance [250] and protects against T-cell senescence [251] via the suppression of insulin-like growth factor 1 and IL-6 [252], as well as decreased mTOR signaling [253] and a decreased anti-OX40 immunotherapy response [254]. Mouse models showed improved immune cell–adipose tissue signaling after CR, with better glucose tolerance and greater fat loss [255]. Moreover, intermittent fasting was correlated with TAM reprogramming, cytotoxic T-cell activation and better anti-tumor immunity [256], with substantial improvements in response when combined with chemotherapy and immunotherapy [257].

##### Gender Effect

Some differences in PDL-1 expression, TMB, LAG-3, and IDO1 were noted between males and females that could affect the response to ICI according to gender type [258]. Many meta-analyses were performed to try to analyze the effect of gender differences on the response to ICI, and some of these studies reported that the overall survival with anti-CTLA-4 treatment could be shorter in females than in males [259,260]. These meta-analyses were done on already published data, so bias control was difficult and interpretation should be performed carefully, as these results could be true for some types of tumors and not others [261,262], and further prospective studies are needed to determine the effect of gender on the response to ICI.

##### Microbiota

The intestinal microbiota has been a hot topic lately since its role in the ICI response has been further investigated; in particular, some studies have reported that the microbiota plays a role in immune surveillance by interacting with tumor antigens and stimulating PRRs to produce cytokines that can have an immunosuppressive or immunostimulatory effect as per the type of cytokine released [263]. The composition and the diversification of the gut microbiota play an important role in the response to ICI as shown by Frankel et al., who reported that patients who had Bacteroidescaccae in their gut flora had better responses to ICI [264]. The use of antibiotics 30 to 60 days prior to treatment with ICI and the use of PPI have been shown to have a deleterious effect on ICI efficacy, as reported by Chalabi et al. [265], and this may be due to the decrease in the diversity of bacterial species and an increase gastric pH, which affects gut microbiomes [266,267]. The gut microbiota and its role in ICI resistance is still not clearly understood, and further studies are needed to further clarify its role. Recent preclinical studies using fecal microbiome transplantation showed an improvement in the response to immunotherapy, and other studies are using microbial ecosystem therapeutics in combination with ICI. Studies are ongoing to try to enhance immunotherapy responses and potentially overcome the resistance to ICI by using the two abovementioned tools [266,268].

## 3. Conclusions

Researchers and clinicians have developed great expectations for immune checkpoint blockade in the field of oncology based on witnessed remarkable patient responses and prolongation of life, with “plateauing” in previously assumed “death-doomed” malignancies. With great enthusiasm for encompassing a larger population of possibly treatable cancers using immunotherapy, significant work has been implemented to turn immunotherapy-resistant or “cold” tumors into immunotherapy-sensitive ones or “hot” tumors, both in the primary and acquired settings. Many mechanisms of resistance—either intrinsic or extrinsic, affecting tumor cells, immune cells or the micro-environment—have been scrutinized in an attempt to counteract them and determine measures to bypass their scope of action, thereby enhancing immune activation and improving patient outcomes. A lot of studies are underway to try to apply these strategies of resisting the resistance to immunotherapy in the clinic, to further the possibility of making advanced cancer a largely treatable disease.

## Figures and Tables

**Table 1:** Mechanisms of resistance to immunotherapy and means to overcome it.

Types	Mechanism of Resistance	Means to Overcome It
Immunosuppressive cells	Tumor-Associated Macrophages (TAMs)	Immune checkpoint inhibitor (ICI )+ CSF1R blockade
	Regulatory T-cells (Tregs)	Anti-PD-1 + Anti-CD25
	Myeloid-derived Suppressor cells (MDSCs)	Anti-CXCR2
	Innate Lymphoid Cells and Tumor Micro-Environment	IL-15, anti-IL-12, anti-IL13, anti-IL-23
Absence of antigen presentation	Beta_2_-Microglobulin (β_2_M)	Adoptive natural killer (NK) cells
	Transporters Associated with Antigen Processing (TAP)	
	Pattern Recognition Receptors (PRR)	PRR agonists
Genetic T-cell exclusion and insensibility to T-cells	MAPK Oncogenic Signaling	MAPK inhibitors
	Tumor Suppressor Phosphate and Tensin Homolog (PTEN)	Anti-PD-1 + Anti-PI3K
	WNT/β -Catenin Signaling Pathway	Anti-PD-1 + BBI608
	Vascular Endothelial Growth Factor (VEGF)	Anti-VEGF + ACT, Anti-VEGF + Anti-CTLA-4
	Transforming Growth Factor- β (TGF-β)	Anti-PD-L1 + Anti- TGF- β
	Indoleamine 2,3-dioxygenase (IDO)	Anti-CTLA-4 + 1MT, IDO inhibitor + ICI, Peptide vaccination + Anti-CTLA-4
	Interferon-gamma Receptor Pathway	Anti-PD-1 + cell-based vaccine
	Enhancer of zester homolog-2 (Ezh2)	Anti-CTLA-4 + GSK503
Impaired T-cell functionality by immunosuppresive signaling receptors	Lymphocyte Activation Gene-3 (LAG-3)	LAG Ig + peptide vaccine, Anti-LAG3 + Anti-PD-1
	T-cell Immunoglobulin Mucin-3 (TIM-3)	Anti-TIM-3 + Anti-PD-1
	V-domain Ig Suppressor of T-cell Activation (VISTA)	Anti-VISTA + peptide cancer vaccine
	T-cell Immunoreceptor with Ig and ITIM domains (TIGIT)	Anti-TIGIT + Anti-PD-1
	B and T Lymphocyte Attenuator-4 (BTLA-4)	Anti-BTLA + Anti-PD-1
Lack of stimulatory checkpoints	ICOS	ICOS stimulators + ICI
	OX40	OX40 stimulators + ICI
	Glucocorticoid-Induced TNF Receptor (GITR)	GITR stimulators + ICI

**Table 2:** Immune checkpoint molecules.

Types	Receptor Partner and Ligands	Action
Stimulatory	ICOS/ICOS-L	Increase T-cell activation and cytotoxicity
	GITR/GITR-L	Increase T-cell activation and decrease Treg cell functions
	CD28/CD80,86	Increase T-cell activation
	CD27/CD70	Increase naïve T-cell proliferation and cytotoxic T-cell differentiation
	CD40/CD40L	Increase T-cell and APC activation
	OX40/OX40-L	Increase T-cell activation and T reg dysfunction/depletion
Inhibitory	PD1/PDL-1	Decrease T-cell activation and increase T reg proliferation
	CTLA-4/CD80,86	Decrease T-cell activation and increase T reg proliferation
	TIM-3/Galectin-9	Decrease T-cell activation and increase T-cell apoptosis and Treg function
	LAG-3/MHC-II	Increase T-cell expansion and T reg cell functions
	VSIG-3/VISTA	Decrease the activity of cytotoxic T-cells, stimulate production of Treg
	BTLA-4/HVEM	Decrease T-cell activation
	TIGIT/CD155, CD112, PVR	Block T-cell activation, increased tolerance of DCs
	CD47/SIRP alpha	Decrease APC presentation

## Abbreviations

APC	Antigen Presenting Cell
ACT	Adoptive cell therapy
β_2_m	Beta_2_-microglobulin
BTLA	B and T lymphocyte attenuator
CTLA-4	Cytotoxic T-lymphocyte-associated protein 4
CSF1	Colony stimulating factor 1
CSF1R	CSF1 receptor
DCs	Dendritic cells
DAMPs	Damage-Associated Molecular Patterns
DNMTi	DNA methyl-transferase Inhibitors
Ezh2	Enhancer of zester Homolog-2
FPR-1	Formyl Peptide Receptor-1
GITR	Glucocorticoid-Induced Tumor Necrosis Factor Receptor
HPD	Hyperprogressive Disease
HPV	Human papillomavirus 16
HVEM	Herpesvirus entry mediator
ICI	Immune checkpoint inhibitor
IDO	Indole-amine 2,3-dioxygenase
Ig	Immunoglobulin
INF	Interferon
IL	Interleukin
ILCs	Innate lymphoid cells
ICOS-L	ICOS-ligand
LAG-3	Lymphocyte Activation Gene-3
MHC-I	Major Histocompatibility Complex-I
MAPK	Mitogen-Activated Protein kinase
MDSCs	Myeloid-Derived Suppressor Cells
NSCLC	Non-small cell lung cancer
NK	Natural killer
NF-κ B	Nuclear factor-κ B
PAMPs	Pathogen-associated molecular patterns
PD-1	Programmed cell death protein-1
PDL-1	Programmed cell death protein ligand-1
PTEN	Tumor Suppressor Phosphatase and Tensin Homolog
PRRs	Pattern Recognition Receptors
PI3K	Phosphatidylinositol 3-kinase
RCC	Renal cell carcinoma
RT	Radiation therapy
STAT1	Signal transducer and activator of transcription 1
TCR	T-cell receptor
Treg	Regulatory T-cells
TSA	Tumor specific antigen
TAMs	Tumor-Associated Macrophages
TILs	Tumor Infiltrating Lymphocytes
TMB	Tumor Mutational Burden
TAP	Transporters Associated with Antigen Processing
TME	Tumor microenvironment
TGF-β	Transforming growth factor beta
TNF-α	Tumor necrosis factor alpha
TIM-3	T-cell Immunoglobulin Mucin-3
TGR	Tumor growth rate
VEGF	Vascular endothelial growth factor
VISTA	V-domain Ig suppressor of T-cell activation
1MT	1-methyl-tryptophan

## References

[1] McCarthy E.F. (2006). The toxins of William B. Coley and the treatment of bone and soft-tissue sarcomas. Iowa Orthop. J..

[2] Ribatti D. (2017). The concept of immune surveillance against tumors. The first theories. Oncotarget.

[3] Dunn G.P., Bruce A.T., Ikeda H., Old L.J., Schreiber R.D. (2002). Cancer immunoediting: From immuno- surveillance to tumor escape. Nat. Immunol..

[4] Vesely M.D., Schreiber R.D. (2013). Cancer immunoediting: Antigens, mechanisms, and implications to cancer immunotherapy. Ann. N. Y. Acad. Sci..

[5] Schumacher T.N., Schreiber R.D. (2015). Neoantigens in cancer immunotherapy. Science.

[6] Ashkenazi R., Gentry S.N., Jackson T.L. (2008). Pathways to tumorigenesis—Modeling mutation acquisition in stem cells and their progeny. Neoplasia.

[7] Kim S. (2015). New and emerging factors in tumorigenesis: An overview. Cancer Manag. Res..

[8] Mittal D., Gubin M.M., Schreiber R.D., Smyth M.J. (2014). New insights into cancer immunoediting and its three component phases—Elimination, equilibrium and escape. Curr. Opin. Immunol..

[9] Gaudino S.J., Kumar P. (2019). Cross-Talk Between Antigen Presenting Cells and T Cells Impacts Intestinal Homeostasis, Bacterial Infections, and Tumorigenesis. Front. Immunol..

[10] Chen L., Flies D.B. (2013). Molecular mechanisms of T cell co-stimulation and co-inhibition. Nat. Rev. Immunol..

[11] Ceeraz S., Nowak E.C., Burns C.M., Noelle R.J. (2014). Immune checkpoint receptors in regulating immune reactivity in rheumatic disease. Arthritis Res. Ther..

[12] Anderson A.C., Joller N., Kuchroo V.K. (2016). Lag-3, Tim-3, and TIGIT: Co-inhibitory Receptors with Specialized Functions in Immune Regulation. Immunity.

[13] Company B.-M.S. (2011). FDA Approves YERVOY™ (ipilimumab) for the Treatment of Patients with Newly Diagnosed or Previously-Treated Unresectable or Metastatic Melanoma, the Deadliest form of Skin Cancer. https://news.bms.com/press-release/rd-news/fda-approves-yervoy-ipilimumab-treatment-patients-newly-diagnosed-or-previousl.

[14] Wei S.C., Anang N.A.A., Sharma R., Andrews M.C., Reuben A., Levine J.H., Allison J.P. (2019). Combination anti-CTLA-4 plus anti-PD-1 checkpoint blockade utilizes cellular mechanisms partially distinct from monotherapies. Proc. Natl. Acad. Sci. USA.

[15] Sun L., Zhang L., Yu J., Zhang Y., Pang X., Ma C., Qiu S. (2020). Clinical efficacy and safety of anti-PD-1/PD-L1 inhibitors for the treatment of advanced or metastatic cancer: A systematic review and meta-analysis. Sci. Rep..

[16] Sharma P., Hu-Lieskovan S., Wargo J.A., Ribas A. (2017). Primary, Adaptive, and Acquired Resistance to Cancer Immunotherapy. Cell.

[17] Pitt J.M., Vétizou M., Daillère R., Roberti M.P., Yamazaki T., Routy B., Zitvogel L. (2016). Resistance Mechanisms to Immune-Checkpoint Blockade in Cancer: Tumor-Intrinsic and -Extrinsic Factors. Immunity.

[18] Yoo S.H., Keam B., Ock C.Y., Kim S., Han B., Kim J.W., Kwon S.K. (2019). Prognostic value of the association between MHC class I downregulation and PD-L1 upregulation in head and neck squamous cell carcinoma patients. Sci. Rep..

[19] García-Aranda M., Redondo M. (2019). Targeting Protein Kinases to Enhance the Response to anti-PD-1/PD-L1 Immunotherapy. Int. J. Mol. Sci..

[20] Ni L., Lu J. (2018). Interferon gamma in cancer immunotherapy. Cancer Med..

[21] Peng W., Chen J.Q., Liu C., Malu S., Creasy C., Tetzlaff M.T., Williams L.J. (2016). Loss of PTEN Promotes Resistance to T Cell-Mediated Immunotherapy. Cancer Discov..

[22] Cheng F., Eng C. (2019). PTEN Mutations Trigger Resistance to Immunotherapy. Trends Mol. Med..

[23] Shin D.S., Zaretsky J.M., Escuin-Ordinas H., Garcia-Diaz A., Hu-Lieskovan S., Kalbasi A., Palaskas N. (2017). Primary Resistance to PD-1 Blockade Mediated by JAK1/2 Mutations. Cancer Discov..

[24] Wang X., Brea L.T., Yu J. (2019). Immune modulatory functions of EZH2 in the tumor microenvironment: Implications in cancer immunotherapy. Am. J. Clin. Exp. Urol..

[25] Trujillo J.A., Luke J.J., Zha Y., Segal J.P., Ritterhouse L.L., Spranger S., Gajewski T.F. (2019). Secondary resistance to immunotherapy associated with β-catenin pathway activation or PTEN loss in metastatic melanoma. J. Immunother. Cancer.

[26] Mouw K.W., Goldberg M.S., Konstantinopoulos P.A., D’Andrea A.D. (2017). DNA Damage and Repair Biomarkers of Immunotherapy Response. Cancer Discov..

[27] De Sousa Linhares A., Leitner J., Grabmeier-Pfistershammer K., Steinberger P. (2018). Not All Immune Checkpoints Are Created Equal. Front. Immunol..

[28] Shitara K., Nishikawa H. (2018). Regulatory T cells: A potential target in cancer immunotherapy. Ann. N. Y. Acad. Sci..

[29] Weber R., Fleming V., Hu X., Nagibin V., Groth C., Altevogt P., Umansky V. (2018). Myeloid-Derived Suppressor Cells Hinder the Anti-Cancer Activity of Immune Checkpoint Inhibitors. Front. Immunol..

[30] Cassetta L., Kitamura T. (2018). Targeting Tumor-Associated Macrophages as a Potential Strategy to Enhance the Response to Immune Checkpoint Inhibitors. Front. Cell Dev. Biol..

[31] Martin-Orozco E., Sanchez-Fernandez A., Ortiz-Parra I., Ayala-San Nicolas M. (2019). WNT Signaling in Tumors: The Way to Evade Drugs and Immunity. Front. Immunol..

[32] Berraondo P., Sanmamed M.F., Ochoa M.C., Etxeberria I., Aznar M.A., Pérez-Gracia J.L., Melero I. (2019). Cytokines in clinical cancer immunotherapy. Br. J. Cancer.

[33] Riaz N., Morris L., Havel J.J., Makarov V., Desrichard A., Chan T.A. (2016). The role of neoantigens in response to immune checkpoint blockade. Int. Immunol..

[34] Kelderman S., Kvistborg P. (2016). Tumor antigens in human cancer control. Biochim. Biophys. Acta.

[35] Yarchoan M., Hopkins A., Jaffee E.M. (2017). Tumor Mutational Burden and Response Rate to PD-1 Inhibition. N. Engl. J. Med..

[36] Keskin D.B., Reinhold B., Lee S.Y., Zhang G., Lank S., O’Connor D., Reinherz E.L. (2011). Direct identification of an HPV-16 tumor antigen from cervical cancer biopsy specimens. Front. Immunol..

[37] Jabbar B., Rafique S., Salo-Ahen O.M., Ali A., Munir M., Idrees M., Rana M.A. (2018). Antigenic Peptide Prediction From E6 and E7 Oncoproteins of HPV Types 16 and 18 for Therapeutic Vaccine Design Using Immunoinformatics and MD Simulation Analysis. Front. Immunol..

[38] Coulie P.G., Van den Eynde B.J., Van Der Bruggen P., Boon T. (2014). Tumour antigens recognized by T lymphocytes: At the core of cancer immunotherapy. Nat. Rev. Cancer.

[39] Yarchoan M., Johnson B.A., Lutz E.R., Laheru D.A., Jaffee E.M. (2017). Targeting neoantigens to augment antitumour immunity. Nat. Rev. Cancer.

[40] Fisk B., Blevins T.L., Wharton J.T., Ioannides C.G. (1995). Identification of an immunodominant peptide of HER-2/neu protooncogene recognized by ovarian tumor-specific cytotoxic T lymphocyte lines. J. Exp. Med..

[41] Rizvi N.A., Hellmann M.D., Snyder A., Kvistborg P., Makarov V., Havel J.J., Miller M.L. (2015). Cancer immunology. Mutational landscape determines sensitivity to PD-1 blockade in non-small cell lung cancer. Science.

[42] Rizvi H., Sanchez-Vega F., La K., Chatila W., Jonsson P., Halpenny D. (2018). Death-Ligand 1 (PD-L1) Blockade in Patients With Non-Small-Cell Lung Cancer Profiled With Targeted Next-Generation Sequencing. J. Clin. Oncol..

[43] Van Allen E.M., Miao D., Schilling B., Shukla S.A., Blank C., Zimmer L., Utikal J. (2015). Genomic correlates of response to CTLA-4 blockade in metastatic melanoma. Science.

[44] Anagnostou V., Smith K., Forde P., Niknafs N., Bhattacharya R., White J., Zhang T., Adleff V., Phallen J., Wali N. (2017). Evolution of neoantigen landscape during immune checkpoint blockade in non-small cell lung cancer. Cancer Discov..

[45] Fares C.M., Van Allen E.M., Drake C.G., Allison J.P., Hu-Lieskovan S. (2019). Mechanisms of Resistance to Immune Checkpoint Blockade: Why Does Checkpoint Inhibitor Immunotherapy Not Work for All Patients?. Am. Soc. Clin. Oncol. Educ. Book.

[46] Spranger S., Luke J.J., Bao R., Zha Y., Hernandez K.M., Li Y., Gajewski A.P., Andrade J., Gajewski T.F. (2016). Density of immunogenic antigens does not explain the presence or absence of the T-cell-inflamed tumor microenvironment in melanoma. Proc. Natl. Acad. Sci. USA.

[47] De Velasco G., Miao D., Voss M., Hakimi A., Hsieh J., Tannir N., Tamboli P., Appleman L., Rathmell W., Van Allen E. (2016). Tumor Mutational Load and Immune Parameters across Metastatic Renal Cell Carcinoma Risk Groups. Cancer Immunol. Res..

[48] Chodon T., Comin-Anduix B., Chmielowski B., Koya R.C., Wu Z., Auerbach M., Ng C., Avramis E., Seja E., Villanueva A. (2014). Adoptive transfer of MART-1 T-cell receptor transgenic lymphocytes and dendritic cell vaccination in patients with metastatic melanoma. Clin. Cancer Res..

[49] Robbins P.F., Kassim S.H., Tran T.L., Crystal J.S., Morgan R.A., Feldman S.A., Yang J.C., Dudley M.E., Wunderlich J.R., Sherry R.M. (2015). A pilot trial using lymphocytes genetically engineered with an NY-ESO-1-reactive T-cell receptor: Long-term follow-up and correlates with response. Clin. Cancer Res..

[50] Robbins P.F., Morgan R.A., Feldman S.A., Yang J.C., Sherry R.M., Dudley M.E., Wunderlich J.R., Nahvi A.V., Helman L.J., Mackall C.L. (2011). Tumor regression in patients with metastatic synovial cell sarcoma and melanoma using genetically engineered lymphocytes reactive with NY-ESO-1. J. Clin. Oncol..

[51] Somaiah N., Block M.S., Kim J.W., Shapiro G.I., Do K.T., Hwu P., Eder J.P., Jones R.L., Lu H., Ter Meulen J.H. (2019). First-in-Class, First-in-Human Study Evaluating LV305, a Dendritic-Cell Tropic Lentiviral Vector, in Sarcoma and Other Solid Tumors Expressing NY-ESO-1. Clin. Cancer Res..

[52] Ott P.A., Hu Z., Keskin D.B., Shukla S.A., Sun J., Bozym D.J., Zhang W., Luoma A., Giobbie-Hurder A., Peter L. (2017). An immunogenic personal neoantigen vaccine for patients with melanoma. Nature.

[53] Moon E.K., Ranganathan R., Eruslanov E., Kim S., Newick K., O’Brien S., Lo A., Liu X., Zhao Y., Albelda S.M. (2016). Blockade of Programmed Death 1 Augments the Ability of Human T Cells Engineered to Target NY-ESO-1 to Control Tumor Growth after Adoptive Transfer. Clin. Cancer Res..

[54] Rosenblatt J., Glotzbecker B., Mills H., Vasir B., Tzachanis D., Levine J.D., Joyce R.M., Wellenstein K., Keefe W., Schickler M. (2011). PD-1 blockade by CT-011, anti-PD-1 antibody, enhances ex vivo T-cell responses to autologous dendritic cell/myeloma fusion vaccine. J. Immunother..

[55] Srivastava P., Paluch B.E., Matsuzaki J., James S.R., Collamat-Lai G., Karbach J., Nemeth M.J., Taverna P., Karpf A.R., Griffiths E.A. (2014). Immunomodulatory action of SGI-110, a hypomethylating agent, in acute myeloid leukemia cells and xenografts. Leuk. Res..

[56] Chiappinelli K.B., Strissel P.L., Desrichard A., Li H., Henke C., Akman B., Hein A., Rote N.S., Cope L.M., Snyder A. (2015). Inhibiting DNA Methylation Causes an Interferon Response in Cancer via dsRNA Including Endogenous Retroviruses. Cell.

[57] Sade-Feldman M., Jiao Y.J., Chen J.H., Rooney M.S., Barzily-Rokni M., Eliane J.-P., Bjorgaard S.L., Hammond M.R., Vitzthum H., Blackmon S.M. (2017). Resistance to checkpoint blockade therapy through inactivation of antigen presentation. Nat. Commun..

[58] Restifo N.P., Marincola F.M., Kawakami Y., Taubenberger J., Yannelli J.R., Rosenberg S.A. (1996). Loss of functional beta 2-microglobulin in metastatic melanomas from five patients receiving immunotherapy. J. Natl. Cancer Inst..

[59] Porgador A., Mandelboim O., Restifo N.P., Strominger J.L. (1997). Natural killer cell lines kill autologous beta2-microglobulin-deficient melanoma cells: Implications for cancer immunotherapy. Proc. Natl. Acad. Sci. USA.

[60] Tao J., Li Y., Liu Y.Q., Li L., Liu J., Shen X., Shen G.X., Tu Y.T. (2008). Expression of transporters associated with antigen processing and human leucocyte antigen class I in malignant melanoma and its association with prognostic factors. Br. J. Dermatol..

[61] Vitale M., Rezzani R., Rodella L., Zauli G., Grigolato P., Cadei M., Hicklin D.J., Ferrone S. (1998). HLA class I antigen and transporter associated with antigen processing (TAP1 and TAP2) down-regulation in high-grade primary breast carcinoma lesions. Cancer Res..

[62] Momburg F., Müllbacher A., Lobigs M. (2001). Modulation of transporter associated with antigen processing (TAP)-mediated peptide import into the endoplasmic reticulum by flavivirus infection. J. Virol..

[63] Turvey S.E., Broide D.H. (2010). Innate immunity. J. Allergy Clin. Immunol..

[64] Ten Broeke T., Wubbolts R., Stoorvogel W. (2013). MHC class II antigen presentation by dendritic cells regulated through endosomal sorting. Cold Spring Harb. Perspect. Biol..

[65] Burgdorf S., Kautz A., Böhnert V., Knolle P.A., Kurts C. (2007). Distinct pathways of antigen uptake and intracellular routing in CD4 and CD8 T cell activation. Science.

[66] Burgdorf S., Kurts C. (2008). Endocytosis mechanisms and the cell biology of antigen presentation. Curr. Opin. Immunol..

[67] Janeway Jr C.A., Medzhitov R. (2002). Innate immune recognition. Annu. Rev. Immunol..

[68] (2006). Reis e Sousa, CDendritic cells in a mature age. Nat. Rev. Immunol..

[69] Kroemer G., Galluzzi L., Kepp O., Zitvogel L. (2013). Immunogenic cell death in cancer therapy. Annu. Rev. Immunol..

[70] Green D.R., Ferguson T., Zitvogel L., Kroemer G. (2009). Immunogenic and tolerogenic cell death. Nat. Rev. Immunol..

[71] Vacchelli E., Ma Y., Baracco E.E., Sistigu A., Enot D.P., Pietrocola F., Yang H., Adjemian S., Chaba K., Semeraro M. (2015). Chemotherapy-induced antitumor immunity requires formyl peptide receptor 1. Science.

[72] Shekarian T., Valsesia-Wittmann S., Brody J., Michallet M., Depil S., Caux C., Marabelle A. (2017). Pattern recognition receptors: Immune targets to enhance cancer immunotherapy. Ann. Oncol..

[73] Ferris R.L., Blumenschein G., Fayette J., Guigay J., Colevas A.D., Licitra L., Harrington K.J., Kasper S., Vokes E.E., Even C. (2018). Nivolumab vs investigator’s choice in recurrent or metastatic squamous cell carcinoma of the head and neck: 2-year long-term survival update of CheckMate 141 with analyses by tumor PD-L1 expression. Oral Oncol..

[74] Ventola C.L. (2017). Cancer Immunotherapy, Part 2: Efficacy, Safety, and Other Clinical Considerations. P T.

[75] Sharabi A.B., Lim M., DeWeese T.L., Drake C.G. (2015). Radiation and checkpoint blockade immunotherapy: Radiosensitisation and potential mechanisms of synergy. Lancet Oncol..

[76] Eriksson D., Stigbrand T. (2010). Radiation-induced cell death mechanisms. Tumour Biol..

[77] Chajon E., Castelli J., Marsiglia H., De Crevoisier R. (2017). The synergistic effect of radiotherapy and immunotherapy: A promising but not simple partnership. Crit. Rev. Oncol. Hematol..

[78] Zhang P., Su D.-M., Liang M., Fu J. (2008). Chemopreventive agents induce programmed death-1-ligand 1 (PD-L1) surface expression in breast cancer cells and promote PD-L1-mediated T cell apoptosis. Mol. Immunol..

[79] Corso C.D., Ali A.N., Diaz R. (2011). Radiation-induced tumor neoantigens: Imaging and therapeutic implications. Am. J. Cancer Res..

[80] Demaria S., Kawashima N., Yang A.M., Devitt M.L., Babb J.S., Allison J.P., Formenti S.C. (2005). Immune-mediated inhibition of metastases after treatment with local radiation and CTLA-4 blockade in a mouse model of breast cancer. Clin. Cancer Res..

[81] Dewan M.Z., Galloway A.E., Kawashima N., Dewyngaert J.K., Babb J.S., Formenti S.C., Demaria S. (2009). Fractionated but not single-dose radiotherapy induces an immune-mediated abscopal effect when combined with anti-CTLA-4 antibody. Clin. Cancer Res..

[82] Kepp O., Galluzzi L., Martins I., Schlemmer F., Adjemian S., Michaud M., Sukkurwala A.Q., Menger L., Zitvogel L., Kroemer G. (2011). Molecular determinants of immunogenic cell death elicited by anticancer chemotherapy. Cancer Metastasis Rev..

[83] Emens L., Middleton G. (2015). The interplay of immunotherapy and chemotherapy: Harnessing potential synergies. Cancer Immunol. Res..

[84] Wang Y.-J., Fletcher R., Yu J., Zhang L. (2018). Immunogenic effects of chemotherapy-induced tumor cell death. Genes Dis..

[85] Liu C., Peng W., Xu C., Lou Y., Zhang M., Wargo J.A., Chen J.Q., Li H.S., Watowich S.S., Yang Y. (2013). BRAF inhibition increases tumor infiltration by T cells and enhances the antitumor activity of adoptive immunotherapy in mice. Clin. Cancer Res..

[86] Loi S., Dushyanthen S., Beavis P.A., Salgado R., Denkert C., Savas P., Combs S., Rimm D.L., Giltnane J.M., Estrada M.V. (2016). RAS/MAPK Activation Is Associated with Reduced Tumor-Infiltrating Lymphocytes in Triple-Negative Breast Cancer: Therapeutic Cooperation Between MEK and PD-1/PD-L1 Immune Checkpoint Inhibitors. Clin. Cancer Res..

[87] Hu-Lieskovan S., Mok S., Moreno B.H., Tsoi J., Robert L., Goedert L., Pinheiro E.M., Koya R.C., Graeber T.G., Comin-Anduix B. (2015). Improved antitumor activity of immunotherapy with BRAF and MEK inhibitors in BRAF(V600E) melanoma. Sci. Transl. Med..

[88] Liu L., Mayes P.A., Eastman S., Shi H., Yadavilli S., Zhang T., Yang J., Seestaller-Wehr L., Zhang S.-Y., Hopson C. (2015). The BRAF and MEK Inhibitors Dabrafenib and Trametinib: Effects on Immune Function and in Combination with Immunomodulatory Antibodies Targeting PD-1, PD-L1, and CTLA-4. Clin. Cancer Res..

[89] Bendell J.C., Kim T.W., Goh B.C., Wallin J., Oh D.-Y., Han S.-W., Lee C.B., Hellmann M.D., Desai J., Lewin J.H. (2016). Clinical activity and safety of cobimetinib (cobi) and atezolizumab in colorectal cancer (CRC). J. Clin. Oncol..

[90] Ribas A., Butler M., Lutzky J., Lawrence D.P., Robert C., Miller W., Linette G.P., Ascierto P.A., Kuzel T., Algazi A.P. (2015). Phase I study combining anti-PD-L1 (MEDI4736) with BRAF (dabrafenib) and/or MEK (trametinib) inhibitors in advanced melanoma. J. Clin. Oncol..

[91] Kaur S., Uddin S., Platanias L.C. (2005). The PI3’ kinase pathway in interferon signaling. J. Interferon Cytokine Res..

[92] Parsa A.T., Waldron J.S., Panner A., Crane C.A., Parney I.F., Barry J.J., Cachola K.E., Murray J.C., Tihan T., Jensen M.C. (2007). Loss of tumor suppressor PTEN function increases B7-H1 expression and immunoresistance in glioma. Nat. Med..

[93] Zhan T., Rindtorff N., Boutros M. (2017). Wnt signaling in cancer. Oncogene.

[94] Spranger S., Bao R., Gajewski T.F. (2015). Melanoma-intrinsic β-catenin signalling prevents anti-tumour immunity. Nature.

[95] Shinozaki E., Kawazoe A., Kuboki Y., Komatsu Y., Nishina T., Hara H., Yuki S., Shitara K., Bando H., Kotani D. (2018). Multicenter phase I/II trial of BBI608 and pembrolizumab combination in patients with metastatic colorectal cancer (SCOOP Study): EPOC1503. J. Clin. Oncol..

[96] Voron T., Colussi O., Marcheteau E., Pernot S., Nizard M., Pointet A.-L., Latreche S., Bergaya S., Benhamouda N., Tanchot C. (2015). VEGF-A modulates expression of inhibitory checkpoints on CD8+ T cells in tumors. J. Exp. Med..

[97] Ohm J.E., Carbone D.P. (2001). VEGF as a mediator of tumor-associated immunodeficiency. Immunol. Res..

[98] Motz G.T., Santoro S.P., Wang L.-P., Garrabrant T., Lastra R.R., Hagemann I.S., Lal P., Feldman M.D., Benencia F., Coukos G. (2014). Tumor endothelium FasL establishes a selective immune barrier promoting tolerance in tumors. Nat. Med..

[99] Shrimali R.K., Yu Z., Theoret M.R., Chinnasamy D., Restifo N.P., Rosenberg S.A. (2010). Antiangiogenic agents can increase lymphocyte infiltration into tumor and enhance the effectiveness of adoptive immunotherapy of cancer. Cancer Res..

[100] Hodi F., Lawrence D., Lezcano C., Wu X., Zhou J., Sasada T., Zeng W., Giobbie-Hurder A., Atkins M., Ibrahim N. (2014). Bevacizumab plus ipilimumab in patients with metastatic melanoma. Cancer Immunol. Res..

[101] Yi M., Jiao D., Qin S., Chu Q., Wu K., Li A. (2019). Synergistic effect of immune checkpoint blockade and anti-angiogenesis in cancer treatment. Mol. Cancer.

[102] Massagué J. (2008). TGFbeta in Cancer. Cell.

[103] Bai X., Yi M., Jiao Y., Chu Q., Wu K. (2019). Blocking TGF-β Signaling To Enhance The Efficacy Of Immune Checkpoint Inhibitor. Onco Targets Ther..

[104] Eberlein C., Kendrew J., McDaid K., Alfred A., Kang J., Jacobs V., Ross S., Rooney C., Smith N., Rinkenberger J. (2013). A human monoclonal antibody 264RAD targeting αvβ6 integrin reduces tumour growth and metastasis, and modulates key biomarkers in vivo. Oncogene.

[105] Morris J.C., Tan A.R., Olencki T.E., Shapiro G.I., Dezube B.J., Reiss M., Hsu F.J., Berzofsky J.A., Lawrence D.P. (2014). Phase I study of GC1008 (fresolimumab): A human anti-transforming growth factor-beta (TGFβ) monoclonal antibody in patients with advanced malignant melanoma or renal cell carcinoma. PLoS ONE.

[106] Mariathasan S., Turley S.J., Nickles D., Castiglioni A., Yuen K., Wang Y., Kadel E.E., Koeppen H., Astarita J.L., Cubas R. (2018). TGFβ attenuates tumour response to PD-L1 blockade by contributing to exclusion of T cells. Nature.

[107] Tolcher A.W., Berlin J.D., Cosaert J., Kauh J., Chan E., Piha-Paul S.A., Amaya A., Tang S., Driscoll K., Kimbung R. (2017). A phase 1 study of anti-TGFβ receptor type-II monoclonal antibody LY3022859 in patients with advanced solid tumors. Cancer Chemother. Pharmacol..

[108] Holmgaard R.B., Zamarin D., Munn D.H., Wolchok J.D., Allison J.P. (2013). Indoleamine 2,3-dioxygenase is a critical resistance mechanism in antitumor T cell immunotherapy targeting CTLA-4. J. Exp. Med..

[109] Van Baren N., Van den Eynde B. (2015). Tryptophan-degrading enzymes in tumoral immune resistance. Front. Immunol..

[110] Bjoern J., Iversen T.Z., Nitschke N.J., Andersen M.H., Svane I.M. (2016). Safety, immune and clinical responses in metastatic melanoma patients vaccinated with a long peptide derived from indoleamine 2,3-dioxygenase in combination with ipilimumab. Cytotherapy.

[111] Zaretsky J.M., Garcia-Diaz A., Shin D.S., Escuin-Ordinas H., Hugo W., Hu-Lieskovan S., Torrejon D.Y., Abril-Rodriguez G., Sandoval S., Barthly L. (2016). Mutations Associated with Acquired Resistance to PD-1 Blockade in Melanoma. N. Engl. J. Med..

[112] Kim Y.J. (2014). Subverting the adaptive immune resistance mechanism to improve clinical responses to immune checkpoint blockade therapy. Oncoimmunology.

[113] Varambally S., Dhanasekaran S.M., Zhou M., Barrette T.R., Kumar-Sinha C., Sanda M.G., Ghosh D., Pienta K.J., Sewalt R.G., Otte A.P. (2002). The polycomb group protein EZH2 is involved in progression of prostate cancer. Nature.

[114] Kim K.H., Roberts C.W. (2016). Targeting EZH2 in cancer. Nat. Med..

[115] Peng D., Kryczek I., Nagarsheth N., Zhao L., Wei S., Wang W., Sun Y., Zhao E., Vatan L., Szeliga W. (2015). Epigenetic silencing of TH1-type chemokines shapes tumour immunity and immunotherapy. Nature.

[116] Zingg D., Arenas-Ramirez N., Sahin D., Rosalia R.A., Antunes A.T., Haeusel J., Sommer L., Boyman O. (2017). The Histone Methyltransferase Ezh2 Controls Mechanisms of Adaptive Resistance to Tumor Immunotherapy. Cell Rep..

[117] Buchbinder E.I., Desai A. (2016). CTLA-4 and PD-1 Pathways: Similarities, Differences, and Implications of Their Inhibition. Am. J. Clin. Oncol..

[118] Rowshanravan B., Halliday N., Sansom D.M. (2018). CTLA-4: A moving target in immunotherapy. Blood.

[119] Hodi F.S., Mihm M.C., Soiffer R.J., Haluska F.G., Butler M., Seiden M.V., Davis T., Henry-Spires R., MacRae S., Willman A. (2003). Biologic activity of cytotoxic T lymphocyte-associated antigen 4 antibody blockade in previously vaccinated metastatic melanoma and ovarian carcinoma patients. Proc. Natl. Acad. Sci. USA.

[120] Phan G.Q., Yang J.C., Sherry R.M., Hwu P., Topalian S.L., Schwartzentruber D.J., Restifo N.P., Haworth L.R., Seipp C.A., Freezer L.J. (2003). Cancer regression and autoimmunity induced by cytotoxic T lymphocyte-associated antigen 4 blockade in patients with metastatic melanoma. Proc. Natl. Acad. Sci. USA.

[121] Robert C., Thomas L., Bondarenko I., O’Day S., Weber J., Garbe C., Lebbe C., Baurain J.-F., Testori A., Grob J.-J. (2011). Ipilimumab plus dacarbazine for previously untreated metastatic melanoma. N. Engl. J. Med..

[122] Antonia S.J., López-Martin J.A., Bendell J., Ott P.A., Taylor M., Eder J.P., Jäger D., Pietanza M.C., Le D.T., de Braud F. (2016). Nivolumab alone and nivolumab plus ipilimumab in recurrent small-cell lung cancer (CheckMate 032): A multicentre, open-label, phase 1/2 trial. Lancet Oncol..

[123] Dosset M., Vargas T.R., Lagrange A., Boidot R., Végran F., Roussey A., Chalmin F., Dondaine L., Paul C., Marie-Joseph E.L. (2018). PD-1/PD-L1 pathway: An adaptive immune resistance mechanism to immunogenic chemotherapy in colorectal cancer. Oncoimmunology.

[124] Wu Y., Chen W., Xu Z.P.G., Gu W. (2019). PD-L1 Distribution and Perspective for Cancer Immunotherapy-Blockade, Knockdown, or Inhibition. Front. Immunol..

[125] Ju X., Zhang H., Zhou Z., Wang Q. (2020). Regulation of PD-L1 expression in cancer and clinical implications in immunotherapy. Am. J. Cancer Res..

[126] Li X., Lian Z., Wang S., Xing L., Yu J. (2018). Interactions between EGFR and PD-1/PD-L1 pathway: Implications for treatment of NSCLC. Cancer Lett..

[127] Ahn M.-J. (2019). Combination of Osimertinib with Durvalumab in Epidermal Growth Factor Receptor-Mutant Non-Small Cell Lung Cancer: Is There Room for Reinvestigation?. J. Thorac. Oncol..

[128] Gettinger S., Hellmann M.D., Chow L.Q., Borghaei H., Antonia S., Brahmer J.R., Goldman J.W., Gerber D.E., Juergens R.A., Shepherd F.A. (2018). Nivolumab Plus Erlotinib in Patients With EGFR-Mutant Advanced NSCLC. J. Thorac. Oncol..

[129] Lin C., Chen X., Li M., Liu J., Qi X., Yang W., Zhang H., Cai Z., Dai Y., Ouyang X. (2015). Programmed Death-Ligand 1 Expression Predicts Tyrosine Kinase Inhibitor Response and Better Prognosis in a Cohort of Patients With Epidermal Growth Factor Receptor Mutation-Positive Lung Adenocarcinoma. Clin. Lung Cancer.

[130] Champiat S., Dercle L., Ammari S., Massard C., Hollebecque A., Postel-Vinay S., Chaput N., Eggermont A., Marabelle A., Soria J.-C. (2017). Hyperprogressive Disease Is a New Pattern of Progression in Cancer Patients Treated by Anti-PD-1/PD-L1. Clin. Cancer Res..

[131] Wang X., Wang F., Zhong M., Yarden Y., Fu L. (2020). The biomarkers of hyperprogressive disease in PD-1/PD-L1 blockage therapy. Mol. Cancer.

[132] Nag S., Zhang X., Srivenugopal K., Wang M.-H., Wang W., Zhang R. (2014). Targeting MDM2-p53 interaction for cancer therapy: Are we there yet?. Curr. Med. Chem..

[133] Kato S., Goodman A., Walavalkar V., Barkauskas D.A., Sharabi A., Kurzrock R. (2017). Hyperprogressors after Immunotherapy: Analysis of Genomic Alterations Associated with Accelerated Growth Rate. Clin. Cancer Res..

[134] Champiat S., Ferrara R., Massard C., Besse B., Marabelle A., Soria J.-C., Ferté C. (2018). Hyperprogressive disease: Recognizing a novel pattern to improve patient management. Nat. Rev. Clin. Oncol..

[135] Sasaki A., Nakamura Y., Mishima S., Kawazoe A., Kuboki Y., Bando H., Kojima T., Doi T., Ohtsu A., Yoshino T. (2019). Predictive factors for hyperprogressive disease during nivolumab as anti-PD1 treatment in patients with advanced gastric cancer. Gastric Cancer.

[136] Peters S., Cappuzzo F., Horn L., Paz-Ares L., Borghaei H., Barlesi F., Steins M., Felip E., Spigel D., Dorange C. (2017). OA03.05 Analysis of Early Survival in Patients with Advanced Non-Squamous NSCLC Treated with Nivolumab vs Docetaxel in CheckMate 057. J. Thorac. Oncol..

[137] Ferrara R., Mezquita L., Texier M., Lahmar J., Audigier-Valette C., Tessonnier L., Mazieres J., Zalcman G., Brosseau S., Le Moulec S. (2018). Hyperprogressive Disease in Patients With Advanced Non-Small Cell Lung Cancer Treated With PD-1/PD-L1 Inhibitors or With Single-Agent Chemotherapy. JAMA Oncol..

[138] Grosso J.F., Goldberg M.V., Getnet D., Bruno T.C., Yen H.-R., Pyle K.J., Hipkiss E., Vignali D.A., Pardoll D.M., Drake C.G. (2009). Functionally distinct LAG-3 and PD-1 subsets on activated and chronically stimulated CD8 T cells. J. Immunol..

[139] Sakuishi K., Apetoh L., Sullivan J.M., Blazar B.R., Kuchroo V.K., Anderson A.C. (2010). Targeting Tim-3 and PD-1 pathways to reverse T cell exhaustion and restore anti-tumor immunity. J. Exp. Med..

[140] Koyama S., Akbay E.A., Li Y.Y., Herter-Sprie G.S., Buczkowski K.A., Richards W.G., Gandhi L., Redig A.J., Rodig S.J., Asahina H. (2016). Adaptive resistance to therapeutic PD-1 blockade is associated with upregulation of alternative immune checkpoints. Nat. Commun..

[141] Lines J.L., Pantazi E., Mak J., Sempere L.F., Wang L., O’Connell S., Ceeraz S., Suriawinata A.A., Yan S., Ernstoff M.S. (2014). VISTA is an immune checkpoint molecule for human T cells. Cancer Res..

[142] Le Mercier I., Chen W., Lines J.L., Day M., Li J., Sergent P., Noelle R.J., Wang L. (2014). VISTA Regulates the Development of Protective Antitumor Immunity. Cancer Res..

[143] Boles K.S., Vermi W., Facchetti F., Fuchs A., Wilson T.J., Diacovo T.G., Cella M., Colonna M. (2009). A novel molecular interaction for the adhesion of follicular CD4 T cells to follicular DC. Eur. J. Immunol..

[144] Stanietsky N., Simic H., Arapovic J., Toporik A., Levy O., Novik A., Levine Z., Beiman M., Dassa L., Achdout H. (2009). The interaction of TIGIT with PVR and PVRL2 inhibits human NK cell cytotoxicity. Proc. Natl. Acad. Sci. USA.

[145] Yu X., Harden K., Gonzalez L.C., Francesco M., Chiang E., Irving B., Tom I., Ivelja S., Refino C.J., Clark H. (2009). The surface protein TIGIT suppresses T cell activation by promoting the generation of mature immunoregulatory dendritic cells. Nat. Immunol..

[146] Huang C., Zhu H.-X., Yao Y., Bian Z.-H., Zheng Y.-J., Li L., Moutsopoulos H.M., Gershwin M.E., Lian Z.-X. (2019). Immune checkpoint molecules. Possible future therapeutic implications in autoimmune diseases. J. Autoimmun..

[147] Burton B.R., Britton G.J., Fang H., Verhagen J., Smithers B., Sabatos-Peyton C.A., Carney L.J., Gough J., Strobel S., Wraith D.C. (2014). Sequential transcriptional changes dictate safe and effective antigen-specific immunotherapy. Nat. Commun..

[148] Joller N., Lozano E., Burkett P.R., Patel B., Xiao S., Zhu C., Xia J., Tan T.G., Sefik E., Yajnik V. (2014). Treg cells expressing the coinhibitory molecule TIGIT selectively inhibit proinflammatory Th1 and Th17 cell responses. Immunity.

[149] Lozano E., Dominguez-Villar M., Kuchroo V., Hafler D.A. (2012). The TIGIT/CD226 axis regulates human T cell function. J. Immunol..

[150] Chauvin J.-M., Pagliano O., Fourcade J., Sun Z., Wang H., Sander C., Kirkwood J.M., Chen T.-H.T., Maurer M., Korman A.J. (2015). TIGIT and PD-1 impair tumor antigen-specific CD8⁺ T cells in melanoma patients. J. Clin. Investig..

[151] Johnston R.J., Comps-Agrar L., Hackney J., Yu X., Huseni M., Yang Y., Park S., Javinal V., Chiu H., Irving B. (2014). The immunoreceptor TIGIT regulates antitumor and antiviral CD8(+) T cell effector function. Cancer Cell.

[152] Fuchs C.S., Özgüroğlu M., Bang Y.-J., Di Bartolomeo M., Mandalà M., Ryu M.-h., Fornaro L., Olesinski T., Caglevic C., Chung H.C. (2020). Pembrolizumab versus paclitaxel for previously treated patients with PD-L1–positive advanced gastric or gastroesophageal junction cancer (GC): Update from the phase III KEYNOTE-061 trial. J. Clin. Oncol..

[153] Watanabe N., Gavrieli M., Sedy J.R., Yang J., Fallarino F., Loftin S.K., Hurchla M.A., Zimmerman N., Sim J., Zang X. (2003). BTLA is a lymphocyte inhibitory receptor with similarities to CTLA-4 and PD-1. Nat. Immunol..

[154] Murphy T.L., Murphy K.M. (2010). Slow down and survive: Enigmatic immunoregulation by BTLA and HVEM. Annu. Rev. Immunol..

[155] Sedy J.R., Gavrieli M., Potter K.G., Hurchla M.A., Lindsley R.C., Hildner K., Scheu S., Pfeffer K., Ware C.F., Murphy T.L. (2005). B and T lymphocyte attenuator regulates T cell activation through interaction with herpesvirus entry mediator. Nat. Immunol..

[156] Cheung T.C., Steinberg M.W., Oborne L.M., Macauley M.G., Fukuyama S., Sanjo H., D’Souza C., Norris P.S., Pfeffer K., Murphy K.M. (2009). Unconventional ligand activation of herpesvirus entry mediator signals cell survival. Proc. Natl. Acad. Sci. USA.

[157] Cai G., Freeman G.J. (2009). The CD160, BTLA, LIGHT/HVEM pathway: A bidirectional switch regulating T-cell activation. Immunol. Rev..

[158] Haymaker C.L., Wu R.C., Ritthipichai K., Bernatchez C., Forget M.-A., Chen J.Q., Liu H., Wang E., Marincola F., Hwu P. (2015). BTLA marks a less-differentiated tumor-infiltrating lymphocyte subset in melanoma with enhanced survival properties. Oncoimmunology.

[159] Stecher C., Battin C., Leitner J., Zettl M., Grabmeier-Pfistershammer K., Höller C., Zlabinger G.J., Steinberger P. (2017). PD-1 Blockade Promotes Emerging Checkpoint Inhibitors in Enhancing T Cell Responses to Allogeneic Dendritic Cells. Front. Immunol..

[160] Fourcade J., Sun Z., Pagliano O., Guillaume P., Luescher I.F., Sander C., Kirkwood J.M., Olive D., Kuchroo V., Zarour H.M. (2012). CD8(+) T cells specific for tumor antigens can be rendered dysfunctional by the tumor microenvironment through upregulation of the inhibitory receptors BTLA and PD-1. Cancer Res..

[161] Pardoll D.M. (2012). The blockade of immune checkpoints in cancer immunotherapy. Nat. Rev. Cancer.

[162] Aicher A., Hayden-Ledbetter M., Brady W.A., Pezzutto A., Richter G., Magaletti D., Buckwalter S., Ledbetter J.A., Clark E.A. (2000). Characterization of human inducible costimulator ligand expression and function. J. Immunol..

[163] Coyle A.J., Lehar S., Lloyd C., Tian J., Delaney T., Manning S., Nguyen T., Burwell T., Schneider H., Gonzalo J.A. (2000). The CD28-related molecule ICOS is required for effective T cell-dependent immune responses. Immunity.

[164] Gigoux M., Lovato A., Leconte J., Leung J., Sonenberg N., Suh W.-K. (2014). Inducible costimulator facilitates T-dependent B cell activation by augmenting IL-4 translation. Mol. Immunol..

[165] Burugu S., Dancsok A.R., Nielsen T.O. (2018). Emerging targets in cancer immunotherapy. Semin. Cancer Biol..

[166] Lee H., Kim J.H., Yang S.Y., Kong J., Oh M., Jeong D.H., Chung J.-I., Bae K.B., Shin J.Y., Hong K.H. (2010). Peripheral blood gene expression of B7 and CD28 family members associated with tumor progression and microscopic lymphovascular invasion in colon cancer patients. J. Cancer Res. Clin. Oncol..

[167] Fan X., Quezada S.A., Sepulveda M.A., Sharma P., Allison J.P. (2014). Engagement of the ICOS pathway markedly enhances efficacy of CTLA-4 blockade in cancer immunotherapy. J. Exp. Med..

[168] Fu T., He Q., Sharma P. (2011). The ICOS/ICOSL pathway is required for optimal antitumor responses mediated by anti-CTLA-4 therapy. Cancer Res..

[169] Kamphorst A.O., Pillai R.N., Yang S., Nasti T.H., Akondy R.S., Wieland A., Sica G.L., Yu K., Koenig L., Patel N.T. (2017). Proliferation of PD-1+ CD8 T cells in peripheral blood after PD-1-targeted therapy in lung cancer patients. Proc. Natl. Acad. Sci. USA.

[170] Donini C., D’Ambrosio L., Grignani G., Aglietta M., Sangiolo D. (2018). Next generation immune-checkpoints for cancer therapy. J. Thorac. Dis..

[171] Croft M. (2010). Control of immunity by the TNFR-related molecule OX40 (CD134). Annu. Rev. Immunol..

[172] Mallett S., Fossum S., Barclay A.N. (1990). Characterization of the MRC OX40 antigen of activated CD4 positive T lymphocytes—A molecule related to nerve growth factor receptor. Embo J..

[173] Ruby C.E., Yates M.A., Hirschhorn-Cymerman D., Chlebeck P., Wolchok J.D., Houghton A.N., Offner H., Weinberg A.D. (2009). Cutting Edge: OX40 agonists can drive regulatory T cell expansion if the cytokine milieu is right. J. Immunol..

[174] So T., Song J., Sugie K., Altman A., Croft M. (2006). Signals from OX40 regulate nuclear factor of activated T cells c1 and T cell helper 2 lineage commitment. Proc. Natl. Acad. Sci. USA.

[175] Polesso F., Weinberg A.D., Moran A.E. (2019). Late-Stage Tumor Regression after PD-L1 Blockade Plus a Concurrent OX40 Agonist. Cancer Immunol. Res..

[176] Alves Costa Silva C., Facchinetti F., Routy B., Derosa L. (2020). New pathways in immune stimulation: Targeting OX40. ESMO Open.

[177] Rogers P.R., Song J., Gramaglia I., Killeen N., Croft M. (2001). OX40 promotes Bcl-xL and Bcl-2 expression and is essential for long-term survival of CD4 T cells. Immunity.

[178] Song J., So T., Cheng M., Tang X., Croft M. (2005). Sustained survivin expression from OX40 costimulatory signals drives T cell clonal expansion. Immunity.

[179] Linch S.N., McNamara M.J., Redmond W.L. (2015). Redmond, OX40 Agonists and Combination Immunotherapy: Putting the Pedal to the Metal. Front. Oncol..

[180] Kjaergaard J., Tanaka J., Kim J.A., Rothchild K., Weinberg A., Shu S. (2000). Therapeutic efficacy of OX-40 receptor antibody depends on tumor immunogenicity and anatomic site of tumor growth. Cancer Res..

[181] Weinberg A.D., Rivera M.-M., Prell R., Morris A., Ramstad T., Vetto J.T., Urba W.J., Alvord G., Bunce C., Shields J. (2000). Engagement of the OX-40 receptor in vivo enhances antitumor immunity. J. Immunol..

[182] Aspeslagh S., Postel-Vinay S., Rusakiewicz S., Soria J.-C., Zitvogel L., Marabelle A. (2016). Rationale for anti-OX40 cancer immunotherapy. Eur. J. Cancer.

[183] Gurney A., Marsters S., Huang A., Pitti R., Mark M., Baldwin D., Gray A., Dowd P., Brush J., Heldens S. (1999). Identification of a new member of the tumor necrosis factor family and its receptor, a human ortholog of mouse GITR. Curr. Biol..

[184] Ronchetti S., Zollo O., Bruscoli S., Agostini M., Bianchini R., Nocentini G., Ayroldi E., Riccardi C. (2004). GITR, a member of the TNF receptor superfamily, is costimulatory to mouse T lymphocyte subpopulations. Eur. J. Immunol..

[185] Schaer D.A., Murphy J.T., Wolchok J.D. (2012). Modulation of GITR for cancer immunotherapy. Curr. Opin. Immunol..

[186] Knee D.A., Hewes B., Brogdon J.L. (2016). Rationale for anti-GITR cancer immunotherapy. Eur. J. Cancer.

[187] Aida K., Miyakawa R., Suzuki K., Narumi K., Udagawa T., Yamamoto Y., Chikaraishi T., Yoshida T., Aoki K. (2014). Suppression of Tregs by anti-glucocorticoid induced TNF receptor antibody enhances the antitumor immunity of interferon-α gene therapy for pancreatic cancer. Cancer Sci..

[188] Lu L., Xu X., Zhang B., Zhang R., Ji H., Wang X. (2014). Combined PD-1 blockade and GITR triggering induce a potent antitumor immunity in murine cancer models and synergizes with chemotherapeutic drugs. J. Transl. Med..

[189] Cohen A.D., Schaer D.A., Liu C., Li Y., Hirschhorn-Cymmerman D., Kim S.C., Diab A., Rizzuto G., Duan F., Perales M.A. (2010). Agonist anti-GITR monoclonal antibody induces melanoma tumor immunity in mice by altering regulatory T cell stability and intra-tumor accumulation. PLoS ONE.

[190] Papadopoulos K.P., Autio K.A., Golan T., Dobrenkov K., Chartash E., Li X.N., Wnek R., Long G.V. (2019). Phase 1 study of MK-4166, an anti-human glucocorticoid-induced tumor necrosis factor receptor (GITR) antibody, as monotherapy or with pembrolizumab (pembro) in patients (pts) with advanced solid tumors. J. Clin. Oncol..

[191] Wynn T.A., Chawla A., Pollard J.W. (2013). Macrophage biology in development, homeostasis and disease. Nature.

[192] Li X., Liu R., Su X., Pan Y., Han X., Shao C., Shi Y. (2019). Harnessing tumor-associated macrophages as aids for cancer immunotherapy. Mol. Cancer.

[193] Zhu Y., Knolhoff B.L., Meyer M.A., Nywening T.M., West B.L., Luo J., Wang-Gillam A., Goedegebuure S.P., Linehan D.C., DeNardo D.G. (2014). CSF1/CSF1R blockade reprograms tumor-infiltrating macrophages and improves response to T-cell checkpoint immunotherapy in pancreatic cancer models. Cancer Res..

[194] Francisco L.M., Salinas V.H., Brown K.E., Vanguri V.K., Freeman G.J., Kuchroo V.K., Sharpe A.H. (2009). PD-L1 regulates the development, maintenance, and function of induced regulatory T cells. J. Exp. Med..

[195] Tanaka A., Sakaguchi S. (2017). Regulatory T cells in cancer immunotherapy. Cell Res..

[196] Vargas F.A., Furness A.J., Solomon I., Joshi K., Mekkaoui L., Lesko M.H., Rota E.M., Dahan R., Georgiou A., Sledzinska A. (2017). Fc-Optimized Anti-CD25 Depletes Tumor-Infiltrating Regulatory T Cells and Synergizes with PD-1 Blockade to Eradicate Established Tumors. Immunity.

[197] Verma A., Mathur R., Farooque A., Kaul V., Gupta S., Dwarakanath B.S. (2019). T-Regulatory Cells in Tumor Progression And Therapy. Cancer Manag. Res..

[198] Shrimali R.K., Ahmad S., Verma V., Zeng P., Ananth S., Gaur P., Gittelman R.M., Yusko E., Sanders C., Robins H. (2017). Concurrent PD-1 Blockade Negates the Effects of OX40 Agonist Antibody in Combination Immunotherapy through Inducing T-cell Apoptosis. Cancer Immunol. Res..

[199] Gabrilovich D.I., Nagaraj S. (2009). Myeloid-derived suppressor cells as regulators of the immune system. Nat. Rev. Immunol..

[200] Yang L., Huang J., Ren X., Gorska A.E., Chytil A., Aakre M., Carbone D.P., Matrisian L.M., Richmond A., Lin P.C. (2008). Abrogation of TGF beta signaling in mammary carcinomas recruits Gr-1+CD11b+ myeloid cells that promote metastasis. Cancer Cell.

[201] Meyer C., Cagnon L., Costa-Nunes C.M., Baumgaertner P., Montandon N., Leyvraz L., Michielin O., Romano E., Speiser D.E. (2014). Frequencies of circulating MDSC correlate with clinical outcome of melanoma patients treated with ipilimumab. Cancer Immunol. Immunother..

[202] Ding A., Routkevitch D., Jackson C., Lim M. (2019). Targeting Myeloid Cells in Combination Treatments for Glioma and Other Tumors. Front. Immunol..

[203] Highfill S.L., Cui Y., Giles A.J., Smith J.P., Zhang H., Morse E., Kaplan R.N., Mackall C.L. (2014). Disruption of CXCR2-mediated MDSC tumor trafficking enhances anti-PD1 efficacy. Sci. Transl. Med..

[204] Rao A., Strauss O., Kokkinou E., Bruchard M., Tripathi K.P., Schlums H., Carrasco A., Mazzurana L., Konya V., Villablanca E.J. (2020). Cytokines regulate the antigen-presenting characteristics of human circulating and tissue-resident intestinal ILCs. Nat. Commun..

[205] Crinier A., Vivier E., Bléry M. (2019). Helper-like innate lymphoid cells and cancer immunotherapy. Semin. Immunol..

[206] Yu Y., Tsang J.C., Wang C., Clare S., Wang J., Chen X., Brandt C., Kane L., Campos L.S., Lu L. (2016). Single-cell RNA-seq identifies a PD-1(hi) ILC progenitor and defines its development pathway. Nature.

[207] Taylor S., Huang Y., Mallett G., Stathopoulou C., Felizardo T.C., Sun M.-A., Martin E.L., Zhu N., Woodward E.L., Elias M.S. (2017). PD-1 regulates KLRG1(+) group 2 innate lymphoid cells. J. Exp. Med..

[208] Mariotti F.R., Quatrini L., Munari E., Vacca P., Moretta L. (2019). Innate Lymphoid Cells: Expression of PD-1 and Other Checkpoints in Normal and Pathological Conditions. Front. Immunol..

[209] Tumino N., Martini S., Munari E., Scordamaglia F., Besi F., Mariotti F.R., Bogina G., Mingari M.C., Vacca P., Moretta L. (2019). Presence of innate lymphoid cells in pleural effusions of primary and metastatic tumors: Functional analysis and expression of PD-1 receptor. Int. J. Cancer.

[210] Vacca P., Pesce S., Greppi M., Fulcheri E., Munari E., Olive D., Mingari M.C., Moretta A., Moretta L., Marcenaro E. (2019). PD-1 is expressed by and regulates human group 3 innate lymphoid cells in human decidua. Mucosal Immunol..

[211] Bal S.M., Golebski K., Spits H. (2020). Plasticity of innate lymphoid cell subsets. Nat. Rev. Immunol..

[212] Ma Z., Li W., Yoshiya S., Xu Y., Hata M., El-Darawish Y., Markova T., Yamanishi K., Yamanishi H., Tahara H. (2016). Augmentation of Immune Checkpoint Cancer Immunotherapy with IL18. Clin. Cancer Res..

[213] Coleman M.F., Cozzo A.J., Pfeil A.J., Etigunta S.K., Hursting S.D. (2020). Cell Intrinsic and Systemic Metabolism in Tumor Immunity and Immunotherapy. Cancers (Basel).

[214] Chang C.-H., Qiu J., O’Sullivan D., Buck M.D., Noguchi T., Curtis J.D., Chen Q., Gindin M., Gubin M.M., Van Der Windt G.J. (2015). Metabolic Competition in the Tumor Microenvironment Is a Driver of Cancer Progression. Cell.

[215] Biswas S.K. (2015). Metabolic Reprogramming of Immune Cells in Cancer Progression. Immunity.

[216] Malinarich F., Duan K., Hamid R.A., Bijin A., Lin W.X., Poidinger M., Fairhurst A.-M., Connolly J.E. (2015). High mitochondrial respiration and glycolytic capacity represent a metabolic phenotype of human tolerogenic dendritic cells. J. Immunol..

[217] Chowdhury P.S., Chamoto K., Kumar A., Honjo T. (2018). PPAR-Induced Fatty Acid Oxidation in T Cells Increases the Number of Tumor-Reactive CD8(+) T Cells and Facilitates Anti-PD-1 Therapy. Cancer Immunol. Res..

[218] Calcinotto A., Filipazzi P., Grioni M., Iero M., De Milito A., Ricupito A., Cova A., Canese R., Jachetti E., Rossetti M. (2012). Modulation of microenvironment acidity reverses anergy in human and murine tumor-infiltrating T lymphocytes. Cancer Res..

[219] Johnston R.J., Su L.J., Pinckney J., Critton D., Boyer E., Krishnakumar A., Corbett M., Rankin A.L., Dibella R., Campbell L. (2019). VISTA is an acidic pH-selective ligand for PSGL-1. Nature.

[220] Gottfried E., Kunz-Schughart L.A., Ebner S., Mueller-Klieser W., Hoves S., Andreesen R., Mackensen A., Kreutz M. (2006). Tumor-derived lactic acid modulates dendritic cell activation and antigen expression. Blood.

[221] Ohashi T., Aoki M., Tomita H., Akazawa T., Sato K., Kuze B., Mizuta K., Hara A., Nagaoka H., Inoue N. (2017). M2-like macrophage polarization in high lactic acid-producing head and neck cancer. Cancer Sci..

[222] Triner D., Shah Y.M. (2016). Hypoxia-inducible factors: A central link between inflammation and cancer. J. Clin. Investig..

[223] Doedens A.L., Stockmann C., Rubinstein M.P., Liao D., Zhang N., DeNardo D.G., Coussens L.M., Karin M., Goldrath A.W., Johnson R.S. (2010). Macrophage expression of hypoxia-inducible factor-1 alpha suppresses T-cell function and promotes tumor progression. Cancer Res..

[224] Noman M.Z., Desantis G., Janji B., Hasmim M., Karray S., Dessen P., Bronte V., Chouaib S. (2014). PD-L1 is a novel direct target of HIF-1α, and its blockade under hypoxia enhanced MDSC-mediated T cell activation. J. Exp. Med..

[225] Clambey E.T., McNamee E.N., Westrich J.A., Glover L.E., Campbell E.L., Jedlicka P., de Zoeten E.F., Cambier J.C., Stenmark K.R., Colgan S.P. (2012). Hypoxia-inducible factor-1 alpha-dependent induction of FoxP3 drives regulatory T-cell abundance and function during inflammatory hypoxia of the mucosa. Proc. Natl. Acad. Sci. USA.

[226] Hanna V.S., Hafez E.A.A. (2018). Synopsis of arachidonic acid metabolism: A review. J. Adv. Res..

[227] Zelenay S., Van Der Veen A.G., Böttcher J.P., Snelgrove K.J., Rogers N., Acton S.E., Chakravarty P., Girotti M.R., Marais R., Quezada S.A. (2015). Cyclooxygenase-Dependent Tumor Growth through Evasion of Immunity. Cell.

[228] Miao J., Lu X., Hu Y., Piao C., Wu X., Liu X., Huang C., Wang Y., Li D., Liu J. (2017). Prostaglandin E(2) and PD-1 mediated inhibition of antitumor CTL responses in the human tumor microenvironment. Oncotarget.

[229] Zhao X., Xu Z., Li H. (2017). NSAIDs Use and Reduced Metastasis in Cancer Patients: Results from a meta-analysis. Sci. Rep..

[230] Dong G., Mao Q., Xia W., Xu Y., Wang J., Xu L., Jiang F. (2016). PKM2 and cancer: The function of PKM2 beyond glycolysis. Oncol. Lett..

[231] Palsson-McDermott E.M., Dyck L., Zasłona Z., Menon D., McGettrick A.F., Mills K.H., O’Neill L.A. (2017). Pyruvate Kinase M2 Is Required for the Expression of the Immune Checkpoint PD-L1 in Immune Cells and Tumors. Front. Immunol..

[232] Watanabe R., Shirai T., Namkoong H., Zhang H., Berry G.J., Wallis B.B., Schaefgen B., Harrison D.G., Tremmel J.A., Giacomini J.C. (2017). Pyruvate controls the checkpoint inhibitor PD-L1 and suppresses T cell immunity. J. Clin. Investig..

[233] Liu W.-R., Tian M.-X., Yang L.-X., Lin Y.-L., Jin L., Ding Z.-B., Shen Y.-H., Peng Y.-F., Gao D.-M., Zhou J. (2015). PKM2 promotes metastasis by recruiting myeloid-derived suppressor cells and indicates poor prognosis for hepatocellular carcinoma. Oncotarget.

[234] Hatfield S.M., Kjaergaard J., Lukashev D., Schreiber T.H., Belikoff B., Abbott R., Sethumadhavan S., Philbrook P., Ko K., Cannici R. (2015). Immunological mechanisms of the antitumor effects of supplemental oxygenation. Sci. Transl. Med..

[235] Linnemann C., Schildberg F.A., Schurich A., Diehl L., Hegenbarth S.I., Endl E., Lacher S., Müller C.E., Frey J., Simeoni L. (2009). Adenosine regulates CD8 T-cell priming by inhibition of membrane-proximal T-cell receptor signalling. Immunology.

[236] Allard B., Pommey S., Smyth M.J., Stagg J. (2013). Targeting CD73 enhances the antitumor activity of anti-PD-1 and anti-CTLA-4 mAbs. Clin. Cancer Res..

[237] Overman M.J., LoRusso P., Strickler J.H., Patel S.P., Clarke S.J., Noonan A.M., Prasanna T., Amin M.A., Nemunaitis J.J., Desai J. (2018). Safety, efficacy and pharmacodynamics (PD) of MEDI9447 (oleclumab) alone or in combination with durvalumab in advanced colorectal cancer (CRC) or pancreatic cancer (panc). J. Clin. Oncol..

[238] De Oliveira Leal V., Mafra D. (2013). Adipokines in obesity. Clin. Chim. Acta.

[239] Turer A., Scherer P. (2012). Adiponectin: Mechanistic insights and clinical implications. Diabetologia.

[240] McArdle M.A., Finucane O.M., Connaughton R.M., McMorrow A.M., Roche H.M. (2013). Mechanisms of obesity-induced inflammation and insulin resistance: Insights into the emerging role of nutritional strategies. Front. Endocrinol. (Lausanne).

[241] Ellulu M.S., Patimah I., Khaza’ai H., Rahmat A., Abed Y. (2017). Obesity and inflammation: The linking mechanism and the complications. Arch. Med. Sci..

[242] Rassy E.E., Ghosn M., Rassy N.A., Assi T., Robert C. (2018). Do immune checkpoint inhibitors perform identically in patients with weight extremes?. Immunotherapy.

[243] Unger R.H. (2004). The hyperleptinemia of obesity-regulator of caloric surpluses. Cell.

[244] Clements V., Long T., Long R., Figley C., Ostrand-Rosenberg S. (2018). Frontline Science: High fat diet and leptin promote tumor progression by inducing myeloid-derived suppressor cells. J. Leukoc. Biol..

[245] Schlesinger S., Siegert S., Koch M., Walter J., Heits N., Hinz S., Jacobs G., Hampe J., Schafmayer C., Nöthlings U. (2014). Postdiagnosis body mass index and risk of mortality in colorectal cancer survivors: A prospective study and meta-analysis. Cancer Causes Control.

[246] Lennon H., Sperrin M., Badrick E., Renehan A.G. (2016). The Obesity Paradox in Cancer: A Review. Curr. Oncol. Rep..

[247] Amptoulach S., Gross G., Kalaitzakis E. (2015). Differential impact of obesity and diabetes mellitus on survival after liver resection for colorectal cancer metastases. J. Surg. Res..

[248] Donnelly D., Bajaj S., Yu J., Hsu M., Balar A., Pavlick A., Weber J., Osman I., Zhong J. (2019). The complex relationship between body mass index and response to immune checkpoint inhibition in metastatic melanoma patients. J. Immunother. Cancer.

[249] Galli G., Corsetto P., Ferrara R., Prelaj A., Proto C., Signorelli D., Zilembo N., De Toma A., Pagani F., Randon G. (2019). Impact of cholesterolemia and body mass index on outcome of metastatic non small cell lung cancer treated with immunotherapy. J. Clin. Oncol..

[250] Turbitt W.J., Xu Y., Sosnoski D.M., Collins S.D., Meng H., Mastro A.M., Rogers C.J. (2019). Physical Activity Plus Energy Restriction Prevents 4T1.2 Mammary Tumor Progression, MDSC Accumulation, and an Immunosuppressive Tumor Microenvironment. Cancer Prev. Res. (Phila).

[251] Messaoudi I., Warner J., Fischer M., Park B., Hill B., Mattison J., Lane M.A., Roth G.S., Ingram D.K., Picker L.J. (2006). Delay of T cell senescence by caloric restriction in aged long-lived nonhuman primates. Proc. Natl. Acad. Sci. USA.

[252] Tang D., Tao S., Chen Z., Koliesnik I.O., Calmes P.G., Hoerr V., Han B., Gebert N., Zörnig M., Löffler B. (2016). Dietary restriction improves repopulation but impairs lymphoid differentiation capacity of hematopoietic stem cells in early aging. J. Exp. Med..

[253] Lee J.-E., Rayyan M., Liao A., Edery I., Pletcher S.D. (2017). Acute Dietary Restriction Acts via TOR, PP2A, and Myc Signaling to Boost Innate Immunity in Drosophila. Cell Rep..

[254] Farazi M., Nguyen J., Goldufsky J., Linnane S., Lukaesko L., Weinberg A.D., Ruby C.E. (2014). Caloric restriction maintains OX40 agonist-mediated tumor immunity and CD4 T cell priming during aging. Cancer Immunol. Immunother..

[255] Fabbiano S., Suárez-Zamorano N., Rigo D., Veyrat-Durebex C., Dokic A.S., Colin D.J., Trajkovski M. (2016). Caloric Restriction Leads to Browning of White Adipose Tissue through Type 2 Immune Signaling. Cell Metab..

[256] Di Biase S., Lee C., Brandhorst S., Manes B., Buono R., Cheng C.-W., Cacciottolo M., Martin-Montalvo A., de Cabo R., Wei M. (2016). Fasting-Mimicking Diet Reduces HO-1 to Promote T Cell-Mediated Tumor Cytotoxicity. Cancer Cell.

[257] Lévesque S., Le Naour J., Pietrocola F., Paillet J., Kremer M., Castoldi F., Baracco E.E., Wang Y., Vacchelli E., Stoll G. (2019). A synergistic triad of chemotherapy, immune checkpoint inhibitors, and caloric restriction mimetics eradicates tumors in mice. Oncoimmunology.

[258] Ye Y., Jing Y., Li L., Mills G.B., Diao L., Liu H., Han L. (2020). Sex-associated molecular differences for cancer immunotherapy. Nat. Commun..

[259] Conforti F., Pala L., Bagnardi V., De Pas T., Martinetti M., Viale G., Gelber R.D., Goldhirsch A. (2018). Cancer immunotherapy efficacy and patients’ sex: A systematic review and meta-analysis. Lancet Oncol..

[260] Wu Y., Ju Q., Jia K., Yu J., Shi H., Wu H., Jiang M. (2018). Correlation between sex and efficacy of immune checkpoint inhibitors (PD-1 and CTLA-4 inhibitors). Int. J. Cancer.

[261] Wang S., Cowley L.A., Liu X.-S. (2019). Sex Differences in Cancer Immunotherapy Efficacy, Biomarkers, and Therapeutic Strategy. Molecules.

[262] Grassadonia A., Sperduti I., Vici P., Iezzi L., Brocco D., Gamucci T., Pizzuti L., Maugeri-Saccà M., Marchetti P., Cognetti G. (2018). Effect of Gender on the Outcome of Patients Receiving Immune Checkpoint Inhibitors for Advanced Cancer: A Systematic Review and Meta-Analysis of Phase III Randomized Clinical Trials. J. Clin. Med..

[263] Zitvogel L., Ayyoub M., Routy B., Kroemer G. (2016). Microbiome and Anticancer Immunosurveillance. Cell.

[264] Frankel A.E., Coughlin L.A., Kim J., Froehlich T.W., Xie Y., Frenkel E.P., Koh A.Y. (2017). Metagenomic Shotgun Sequencing and Unbiased Metabolomic Profiling Identify Specific Human Gut Microbiota and Metabolites Associated with Immune Checkpoint Therapy Efficacy in Melanoma Patients. Neoplasia.

[265] Chalabi M., Cardona A., Nagarkar D.R., Dhawahir Scala A., Gandara D.R., Rittmeyer A., Albert M.L., Powles T., Kok M., Herrera F.G. (2020). Efficacy of chemotherapy and atezolizumab in patients with non-small-cell lung cancer receiving antibiotics and proton pump inhibitors: Pooled post hoc analyses of the OAK and POPLAR trials. Ann. Oncol..

[266] Gopalakrishnan V., Spencer C.N., Nezi L., Reuben A., Andrews M.C., Karpinets T.V., Prieto P.A., Vicente D., Hoffman K., Wei S.C. (2018). Gut microbiome modulates response to anti-PD-1 immunotherapy in melanoma patients. Science.

[267] Jackson M.A., Goodrich J.K., Maxan M.-E., Freedberg D.E., Abrams J.A., Poole A.C., Sutter J.L., Welter D., Ley R.E., Bell J.T. (2016). Proton pump inhibitors alter the composition of the gut microbiota. Gut.

[268] Matson V., Fessler J., Bao R., Chongsuwat T., Zha Y., Alegre M.-L., Luke J.J., Gajewski T.F. (2018). The commensal microbiome is associated with anti-PD-1 efficacy in metastatic melanoma patients. Science.

